# Donor aid mentioning newborns and stillbirths, 2002–19: an analysis of levels, trends, and equity

**DOI:** 10.1016/S2214-109X(23)00378-9

**Published:** 2023-10-17

**Authors:** Meghan Bruce Kumar, David Bath, Peter Binyaruka, Jacob Novignon, Joy E Lawn, Catherine Pitt

**Affiliations:** a Department of Global Health and Development, London School of Hygiene & Tropical Medicine, London, UK; b MARCH Centre, London School of Hygiene & Tropical Medicine, London, UK; c KEMRI-Wellcome Trust Programme, Nairobi, Kenya; d Ifakara Health Institute, Dar Es Salaam, Tanzania; e Department of Economics, Kwame Nkrumah University of Science and Technology, Kumasi, Ghana

## Abstract

**Background:**

Global aid for reproductive, maternal, newborn, and child health has stagnated in recent years, and aid mentioning newborns or stillbirths has previously represented a very small proportion of aid for reproductive, maternal, newborn, and child health. Neonatal survival targets have been set by 78 countries, and stillbirth prevention targets have been set by 30 countries, to address the 4·4 million newborn deaths and stillbirths globally. We aimed to generate novel estimates of current levels of, and trends in, aid mentioning newborns and stillbirths over 2002–19, and to assess whether the amount of aid disbursed aligns with the associated mortality burden.

**Methods:**

For this analysis, we did a manual review and coding of the Organisation for Economic Co-operation and Development (OECD)'s Creditor Reporting System database from 2002 to 2019 using key search terms for aid mentioning newborns and stillbirths. We compared these findings with estimates of aid for reproductive, maternal, newborn, and child health for 2002–19 based on the Muskoka2 method. Findings are presented in 2019 US$ according to the OECD's Development Assistance Committee deflators, which account for variation in exchange rates and inflation in donor countries.

**Findings:**

We identified 21 957 unique records in the 2002–19 period. Aid mentioning newborns and stillbirths comprised approximately 10% ($1·6 billion) of reproductive, maternal, newborn, and child health funding overall in 2019 ($15·9 billion), with a small decrease in value between 2015 and 2019. 1284 (6%) of 21 957 records and 3·4% ($535 million) of their total value mentioned aid focused only on newborn health. Ten donors contributed 87% ($13·7 billion) of the total value of aid mentioning newborns and stillbirths during 2002–19. Aid mentioning newborns and stillbirths was inequitably allocated in the least developed countries (as defined by the UN), ranging from $18 per death in Angola to $1389 per death in Timor-Leste. Stillbirths were not mentioned in any funding in 2002–09, and they were only mentioned in 46 of 21 957 records in 2010–19, comprising $44·4 million of aid disbursed during this period.

**Interpretation:**

Aid mentioning newborns and stillbirths is poorly matched to their corresponding mortality burden (representing 10% of aid for reproductive, maternal, newborn, and child health overall, yet accounting for approximately 50% of mortality in children <5 years) and across recipient countries (with substantial variation in the amount of aid received per newborn death and stillbirth between countries with similar health and economic needs). Our findings indicate that aid needs to be better targeted to populations with the highest mortality burdens, creating greater potential for impact.

**Funding:**

John D and Catherine T MacArthur Foundation, Bill & Melinda Gates Foundation, ELMA Philanthropies, Children's Investment Fund Foundation UK, Lemelson Foundation, and Ting Tsung and Wei Fong Chao Foundation.

**Translation:**

For the French translation of the abstract see Supplementary Materials section.

## Introduction

Global reductions in maternal and child mortality in past decades have been impressive,^1^, ^2^, ^3^, ^4^ and child mortality is one of the few UN Sustainable Development Goals (SDGs) on track to be achieved by 2030 in most countries.^5^ By contrast, neonatal mortality (defined as deaths 0–28 days after birth) has been relatively stagnant, with 2·4 million deaths globally in 2020, accounting for nearly 50% of mortality in children younger than 5 years.^6^ Neonatal conditions continued to top the list of conditions leading to most disability-adjusted life-years in all regions in 2019, consistent across 30 years of measurement from baseline in 1990.^7^, ^8^, ^9^, ^10^, ^11^ Stillbirths (defined as deaths after 28 weeks of pregnancy) account for almost 2 million deaths annually.^12^ Policy commitments in the past decade demonstrate increased visibility for newborns and stillbirths, including the first global goal for newborn survival (SDG target 3.2), and the Every Newborn Action Plan (ENAP) ratified by the 2014 World Health Assembly, which included a target to reduce the rate of stillbirths.^3^, ^13^, ^14^ Neonatal survival and stillbirth prevention are seen as tractable, with the possibility of millions of deaths averted through interventions along the continuum of reproductive, maternal, newborn, and child health.^13^ Neonatal survival targets have been set by 78 countries and stillbirth prevention targets have been set by 30 countries to address the 4·4 million newborn deaths and stillbirths globally; more than 106 countries have come together to track and address this challenge at a national level through country-focused ENAPs,^3^, ^13^ with coordinated leadership by WHO and UNICEF.^15^ Achieving these targets and fulfilling these action plans depends on the availability of adequate financing and other resources, particularly in the face of the COVID-19 pandemic and economic shocks around the world.

**Research in context**

**Evidence before this study**

Previous analyses have shown increasing aid funding for maternal, newborn, and child health between 2003 and 2013, totalling more than US$70 billion over this period, of which only 13% mentioned newborns in 2013. Despite the large number of stillbirths (similar to the number of newborn deaths), relevant terms occurred only nine times among these 2·1 million donor disbursement records. In recent years, funding for reproductive, maternal, newborn, and child health appears to be stagnant or falling, possibly exacerbated by the COVID-19 pandemic. At the same time, newborn health has received increasing policy attention, including the first global target for neonatal mortality reduction in the UN Sustainable Development Goals (target 3.2), with momentum in many high-burden countries related to the targets outlined in the *Lancet* Every Newborn Series published in 2014. Although a systematic literature search was not conducted before undertaking this analysis, searches of PubMed revealed only two published multi-country analyses of donor funding for newborns or stillbirths, both of which were undertaken by our team, and the last one was published in 2017, covering data up to the year 2013. Since then, recent advances have been made in assessing donor aid for reproductive, maternal, newborn, and child health with the Muskoka2 method, although more granular categorisation still involves greater uncertainty.

**Added value of this study**

Building on these previous studies, the present analysis extended this work to also assess funding mentioning stillbirths, looking in detail at the share of aid allocated to stillbirths, funders, and recipients. We assessed official development assistance and private grants (together termed aid) mentioning newborn health and stillbirths using the Organisation for Economic Co-operation and Development's Creditor Reporting System database for all years for which donors have reported disbursements (2002–19), with a combination of the Muskoka2 methods, key term searches, and manual coding. Despite almost 90 million newborn deaths and stillbirths over the study period (based on UN estimates of >4·4 million deaths worldwide for the study period), we found that relevant terms including newborn health were mentioned in records valued at just approximately 10% ($1·6 billion) of total aid for reproductive, maternal, newborn, and child health in 2019. Notably, just 3·4% of this proportion ($535 million; ie, 0·4% of aid for reproductive, maternal, newborn, and child health) named interventions that are newborn focused. Out of a total of 21 957 records in 2002–19 identified through the database search, only 46 specifically mentioned stillbirths, with a total value of $44·4 million (constant 2019 US$). Despite the apparently increasing policy traction, the value of global aid mentioning newborns stagnated or decreased. In 2019, $1·6 billion in aid mentioned newborns, compared to a peak of $1·8 billion in 2017. The majority of funding mentioning newborns and stillbirths was disbursed by a small number of donors: in 2019, five bilateral donors disbursed 77% ($12·2 billion) and ten donors disbursed 87% ($13·7 billion) of the total aid. The aid disbursed was broadly aligned with burden and need but inconsistently targeted; aid per newborn death or stillbirth (combined) ranged between $18 (Angola) and $1389 (Timor-Leste) across the 41 least developed countries (as defined by the UN).

**Implications of all the available evidence**

Given that major funding for reproductive, maternal, newborn, and child health is estimated at $15·9 billion per year, more data on aid allocations are required to drive accountability. Assessments of the equity of investments relative to the mortality burden across countries are crucial. In this analysis, we show that overall aid for reproductive, maternal, newborn, and child health still comprises very low amounts mentioning newborns and almost none mentioning stillbirths, despite more than 4·4 million newborn deaths and stillbirths per year and high potential for saving lives. Marked variability in funding per newborn death and stillbirth across countries presents an opportunity to improve targeting of aid for these vulnerable groups in countries with the highest burdens. Domestic funding for the health of these vulnerable groups is more complex to track, but in many countries it exceeds external aid; advancing national tracking is therefore also fundamental for accountability and accelerating progress.

In many low-income and middle-income countries, efforts to improve the quality of and access to reproductive, maternal, newborn, and child health care remain reliant on external financing, with aid representing a median of 22% of reproductive, maternal, newborn, and child health expenditure in 2018 across 23 reporting countries.^16^ In 2019, aid for reproductive, maternal, newborn, and child health was approximately US$15·9 billion globally, 46% of which was estimated to benefit child health—dominated by funding for vaccines and infectious diseases—and 19% was estimated to benefit maternal and newborn health.^16^ Previous work has shown that only 13% of aid for maternal, newborn, and child health mentioned newborns in the 2002–13 period and stillbirths were almost entirely neglected, with only nine mentions in 2·1 million records.^17^ The fragmentation of donors in individual countries and misalignment of aid with country strategies for reproductive, maternal, newborn, and child health might restrict the effectiveness of aid.^18^ Furthermore, ensuring that aid is targeted to countries with the greatest needs is crucial to optimise the impact of any funds disbursed.

Tracking aid is essential to accountability. Although investments to improve newborn health and stillbirth prevention could be categorised under maternal or child health, it is important to examine funding for the specific interventions that benefit these groups (eg, Kangaroo Mother Care, small and sick newborn care, and intra-uterine growth screening) that are not automatically a part of maternal or child health care. We aimed to analyse current levels of and trends in aid mentioning newborns and stillbirths over the 2002–19 period. We compared these analyses with Muskoka2 estimates of aid for reproductive, maternal, newborn, and child health,^16^ aiming to generate evidence to hold donors accountable for commitments by assessing whether aid increased over time and whether the amount of aid disbursed aligns with the corresponding burden of mortality.

## Methods

### Data sources

The Organisation for Economic Co-operation and Development (OECD)'s Creditor Reporting System (CRS) database is used to track aid flows globally on an annual basis. Donor countries, multilateral institutions (eg, UNICEF, WHO), and private foundations report aid activities to the CRS by calendar year, generally with at least a year's delay between the close of a year and the reporting of that year's aid. For each aid activity, the CRS provides data on 87 variables, which include the donor country or organisation, as well as the recipient, purpose, monetary value, year, flow type, and free-text descriptions of the disbursement. In the CRS, the donor is defined as the organisation that retains control over the recipient and purpose of disbursement; accordingly, funding flows through multilateral institutions are categorised as being disbursed either from that multilateral institution or from a bilateral donor if the latter retained control over the specific use of funds. In 2019, 49 donor countries, 46 multilateral institutions, and 37 private institutions reported a non-zero disbursement to the CRS; many of these donors only reported their disbursements in later years of the study period (2002–19). Aid flows to one of 144 recipient countries and 23 recipient regions in the CRS. The CRS uses purpose codes to categorise the “specific area of the recipient's economic or social structure” for which the aid is intended.^19^ There is no purpose code specific to newborns or stillbirths, or both combined.

### Estimation of aid mentioning newborns and stillbirths

**Figure 1:**
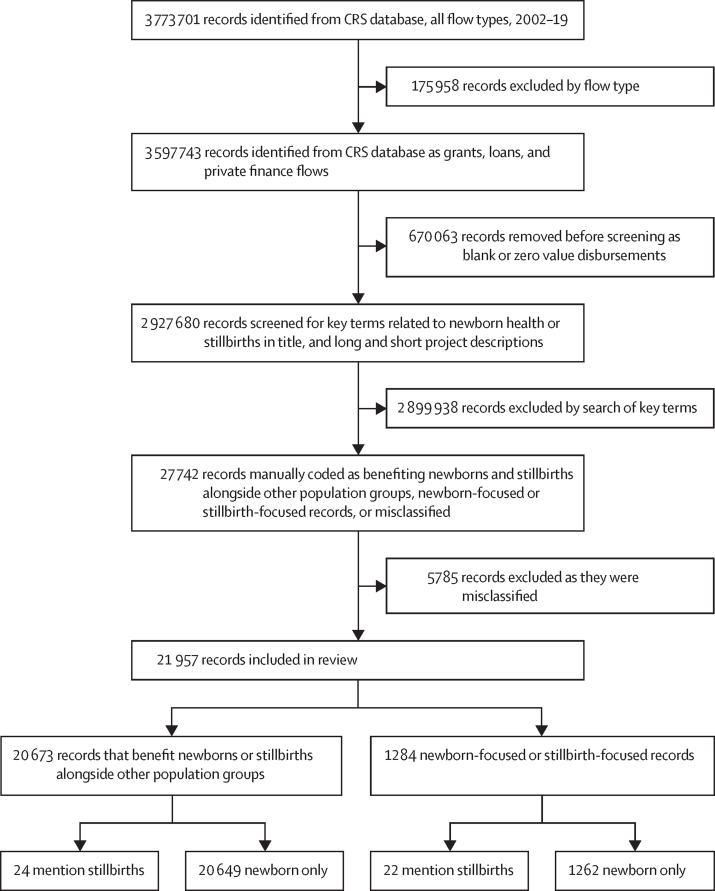
Flow diagram for identification of records mentioning newborn health or stillbirths CRS=Organisation for Economic Co-operation and Development Creditor Reporting System.

Figure 1 illustrates the identification of CRS disbursement records for 2002–19. For this analysis, we included official development assistance (ODA) grants and loans, as well as private development finance, and excluded equity investments and other official flows, consistent with previous research,^17^, ^20^ and aligned with the Muskoka2 method. Records with zero or blank disbursement values were excluded.

We searched each record's project title and short and long project descriptions for key terms to identify funding of interventions in pregnancy or the first month of life that mentioned newborn or stillbirth terms, or included specific items expected to prevent stillbirths or improve neonatal health (appendix 2 p 2). Key search terms used in a previous analysis by Pitt and colleagues^17^ of aid funding from 2003 to 2013 were applied with minor modifications; search terms were removed if they were neither present in this previous analysis nor in a search of the 2019 CRS database, and the term “continuous positive airway pressure” was added (appendix 2 p 3). The search included terms in the seven most common languages in the CRS (Dutch, English, French, German, Italian, Portuguese, and Spanish).

For coding, we collapsed records identified by the key-term search with the same purpose code and short and long project descriptions into a single unique record. We then manually coded all unique records containing any newborn or stillbirth terms into two main categories: misclassified (not relating to human newborn health or stillbirths) or aid mentioning newborns or stillbirths. The second category was further subcategorised as follows: newborn- or stillbirth-focused (no other population groups or outcomes included); or including both newborns or stillbirths and other population groups (eg, the wider reproductive, maternal, newborn, and child health continuum; appendix 2 p 6). The terminology used here (newborn or stillbirth focused) represents a change from previous work by Pitt and colleagues^17^ using the terminology “exclusively benefiting” but represents the same category. This change was made to acknowledge that interventions benefiting newborns often benefit children, women, and families.

DB reviewed and coded all unique records (n=27 742; figure 1) and flagged 1328 for review. MBK reviewed and coded all unique flagged records, as well as those with a value higher than $7 million (528 records) and a random 5% sample (464 records; total 2174 records, categories were not mutually exclusive). MBK flagged 212 records for a third review. CP reviewed records flagged by MBK plus 305 records with discordant codes. All coding was conducted in a blinded format to the other reviewers’ coding. Finally, 37 records where some or all three reviewers disagreed were discussed and a consensus view reached.

Newborn and stillbirth records were categorised as funding for stillbirths if they included the search term “stillb*”; the remainder were categorised as funding for newborns (figure 1).

### Estimation of aid for reproductive, maternal, newborn, and child health

To contextualise the funding mentioning newborns and stillbirths, we compared our findings to Muskoka2 estimates of aid for maternal, newborn, and child health and reproductive, maternal, newborn, and child health for 2002–19.^16^ Muskoka2 is an algorithm applied to the CRS database, which generates estimates of aid for the reproductive health of non-pregnant women, aid for maternal and newborn health, and aid for child health, which can be summed together to generate aggregate estimates of aid for reproductive, maternal, newborn, and child health.^20^ Developed through a stakeholder consultation process, Muskoka2 includes both aid categorised within the CRS's reproductive health and family planning purpose codes, as well as relevant shares of aid directed towards wider activities, including health-system strengthening, infectious disease control, water and sanitation, and humanitarian aid. The Muskoka2 algorithm generates estimates on the basis of purpose codes for aid from most donors, and additionally takes a fixed percentage of disbursements from three multilateral organisations (GAVI, the Vaccine Alliance; UN Population Fund; and UNICEF) with specialised mandates for reproductive, maternal, newborn, and child health.^20^, ^21^ For replicability, full details of the approach are summarised in the report by Dingle and colleagues^20^ and the Muskoka2 estimates for 2002–19 were published by Pitt and colleagues.^16^

### Data analysis

For each included record, we extracted data on flow type, purpose code, donor, recipient, year, and value of project. First, we analysed trends in overall amounts of aid disbursed annually. This was done separately for aid mentioning newborns and stillbirths. We examined annual aid values in the context of estimated aid for maternal, newborn, and child health and for reproductive, maternal, newborn, and child health. We also explored trends by individual donors and donor types (bilateral, multilateral, and private), recipients (all, by region, and least developed countries [as defined by the UN]), and purpose codes.

To assess equity in disbursement, we examined the relationship between disbursed amounts of ODA and health need using three measures of need. First, we selected only least developed countries as defined by the UN.^22^ Then, we ranked recipient countries on the basis of the most recent estimates of the mortality burden from 2002 to 2019, summing neonatal mortality and stillbirths,^6^, ^12^ and compared that order with the amount of aid disbursed to each recipient country included in our analysis. We also divided the amount of aid provided by the total number of deaths to obtain an average aid amount, in US$, per death (appendix 2 p 32).

Findings are presented in 2019 US$ according to the OECD's Development Assistance Committee deflators, which account for variation in exchange rates and inflation in donor countries. For some sub-analyses examining trends over time, we show both all reported data, as well as findings from a restricted set of donors that reported non-zero values in both 2002 and 2019, in order to avoid bias, as more donors reported their disbursements in later years. Aid for regional recipients was not allocated across countries in the region in this analysis due to uncertainties in the allocation.

### Role of the funding source

The funders of the study had no role in study design, data collection, data analysis, data interpretation, writing of the manuscript, or the decision to submit the manuscript for publication.

## Results

We identified 27 742 records that mentioned any newborn or stillbirth terms, comprising 9280 records with a unique project title and project descriptions. After exclusion of 5785 misclassified records, we included 21 957 unique records in the 2002–19 period (figure 1).

**Figure 2:**
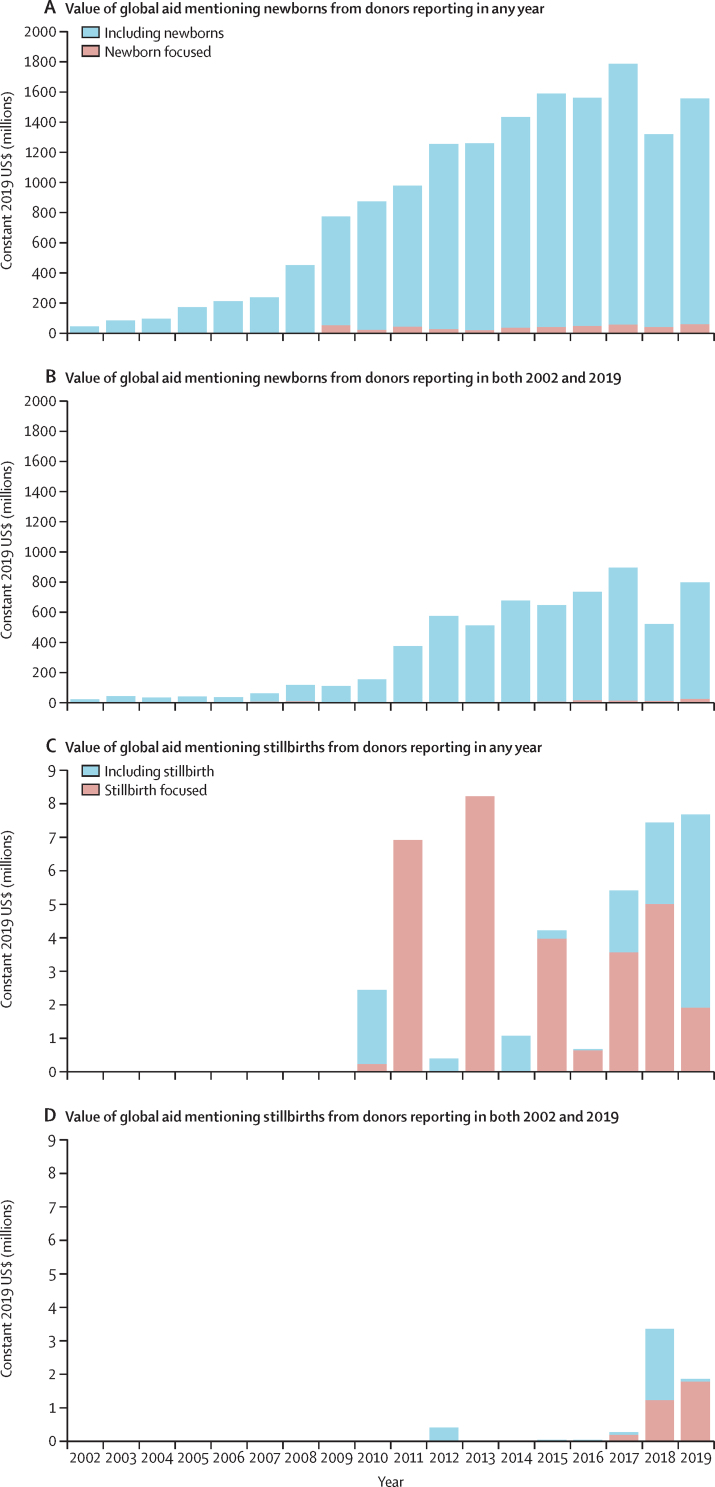
Trends in global aid mentioning and focused on newborns and on stillbirths for 2002–19 Global aid including and focused on newborns for donors reporting in any year (A) and in both 2002 and 2019 (B). Global aid including and focused on stillbirths reporting in any year (C) and in both 2002 and 2019 (D). Not all donors in the database reported in every year, and the number of donors reporting has increased over time. As such, the donors reporting in any year figure shows a more complete list of items mentioning newborns and stillbirths. However, it is possible that those that reported only in later years did fund these health areas in earlier years but those funds were not reported and thus are not captured in this analysis.

Stillbirths remain almost absent in reported aid and were not mentioned in any funding for the years 2002–09. Over the 2010–19 period, only 46 records ($44·4 million) specifically mentioned stillbirths (appendix 2 pp 9–27). Most (69%) of the value of aid mentioning stillbirths was stillbirth focused ($30·5 million), with the remainder ($13·9 million) mentioning stillbirths along with other population groups (figure 2). Values fluctuated over time, with no clear trend; aid mentioning stillbirths peaked in 2013 at $8·2 million.

The total value of the records mentioning newborn health (not stillbirths) was $15*·*7 billion over the 18-year study period (figure 2). The value of global aid mentioning newborns consistently increased, from $49 million in 2002 to a peak of $1·8 billion in 2017, followed by a reduction to $1·3 billion in 2018 (just above the 2013 value), and a rebound to $1·6 billion in 2019.

Most disbursements mentioning newborns did so within projects that would also benefit other population groups, often as part of the term “maternal, newborn, and child health”, without noting any focused interventions. 1284 (6%) of 21 957 records and 3·4% ($535 million) of the value of records mentioning newborns were newborn focused (figure 2). Annual newborn-focused aid was lower than $10 million until 2009 and ranged between $25 million (2013) and $64 million (2017) in 2009–19, with no clear trend in values and relatively little change over time.

**Figure 3:**
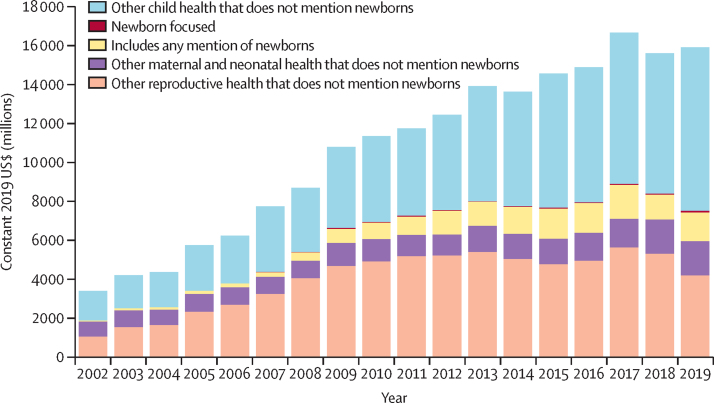
Trends in global aid mentioning newborns in the context of aid for reproductive, maternal, newborn, and child health, 2002–19 The manually coded estimates for global aid mentioning newborns are shown with Muskoka2 estimates of reproductive, maternal, and child health (which do not mention newborns and stillbirths). This figure includes all donors reporting over any subset of the 2002–19 period.

Aid mentioning newborns and stillbirths increased from 2% of aid for maternal, newborn, and child health in 2002 ($49 million) to a high of 17% ($1·4 billion) in 2012, before declining to 13% of aid for maternal, newborn, and child health in 2018 ($1·3 billion) and 2019 ($1·6 billion; figure 3; appendix 2 p 8). When funding for reproductive health is added, in 2019 funding mentioning newborns and stillbirths represented 10% of aid for reproductive, maternal, newborn, and child health overall, a proportion that remained consistently between 8% and 11% from 2009 to 2019. The value of aid mentioning stillbirths remained a tiny fraction of aid to the sector, at 0·04% of aid for maternal, newborn, and child health, and 0·02% of aid for reproductive, maternal, newborn, and child health.

**Figure 4:**
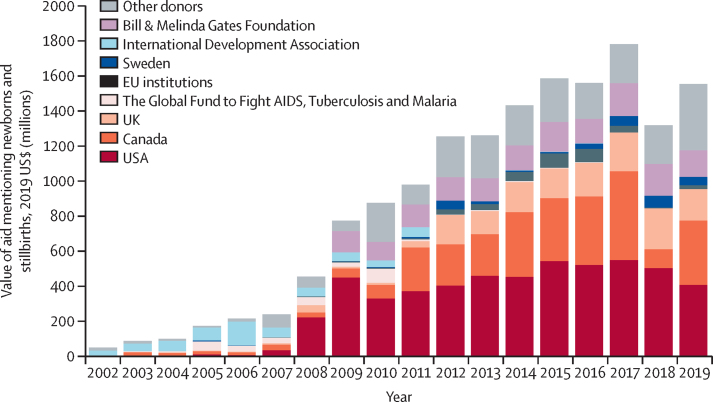
Trends in global aid mentioning newborns by donor, 2002–19

Five donors disbursed 77% of aid ($12·2 billion of $15·2 billion) mentioning newborns and stillbirths, and ten donors disbursed 87% ($13·7 billion) of the aid over 2002–19 (figure 4). Bilateral donors provided most of the aid (75%, $11·8 billion); the USA and Canada alone disbursed about 50% of funding in 2011–19. Multilateral funds for newborn health and stillbirth prevention reduced in importance; the International Development Association–World Bank was an early funder for newborn health, although it disbursed very small absolute values, and disappeared almost completely after 2011. The Bill & Melinda Gates Foundation was the only private donor in the top ten donors. The top funders for aid mentioning stillbirths were Sweden (18 records; $2·7 million) and the Gates Foundation (13 records; $24·9 million), which accounted for 62% of the funding mentioning stillbirths ($27·6 million of $44·6 million; appendix 2 p 9).

Over 2002–19, donors directed 39% ($6·1 billion of $15·2 billion) of the value of aid mentioning newborns and stillbirths towards the reproductive health purpose code, with the remainder directed towards basic health care (10%, $1·7 billion), basic nutrition (7%; $1·1 billion), control of sexually transmitted diseases including HIV/AIDS (6%, $1·0 billion), family planning (5%, $0·8 billion), health policy and administrative management (4%; $0·6 billion), infectious disease control (4%; $0·6 billion), and other purpose codes (<2%; $0·3 billion; appendix 2 p 28). All of these codes fall broadly under the health sector.

The top country recipient of aid mentioning newborns and stillbirths was Ethiopia ($821 million). The top ten country recipients (six in Africa and four in Asia) received 42% of the total value of aid ($15·2 billion) during the 2002–19 period. Ethiopia ($821 million), Pakistan ($790 million), and Kenya ($730 million) received 5% each; Nigeria ($707 million), India ($700 million), Bangladesh ($688 million), Afghanistan ($663 million), and Tanzania ($652 million) received 4% each; and Zimbabwe ($444 million) and Mozambique ($397 million) received 3% each (appendix 2 p 31). The only country in the Americas among the top 30 recipients was Honduras (29th; 1% of funding; $147 million). Most aid (79%; $12·4 billion) went to recipient countries, 8% ($1·0 billion) went to regional recipients, and 14% ($2·3 billion) went to “bilateral unspecified” recipients.

**Figure 5:**
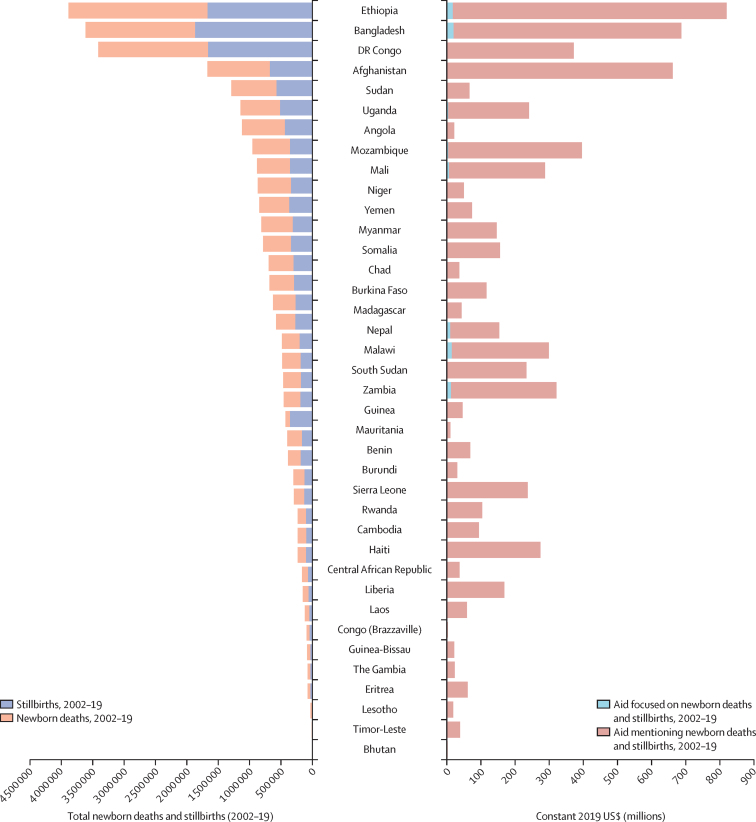
Equity assessment comparing amount of aid received by recipient country against need (burden of newborn deaths and stillbirths, 2002–19) The 41 least developed countries (as defined by the UN) with more than 10 000 births annually are shown, and the countries are ordered by the size of their cumulative burden of stillbirths and newborn deaths in 2002–19 on the left of the graph. Aid mentioning newborns or stillbirths, or both combined, and focusing on newborns and stillbirths is displayed on the right. Source of neonatal and stillbirth deaths: UNICEF, 2023.

The 41 least developed countries (as defined by the UN) collectively received $6·5 billion in aid mentioning newborns and stillbirths over the study period. The value of aid mentioning newborns and stillbirths for each of these countries was broadly aligned with the combined burden of the number of neonatal deaths and stillbirths, but with substantial variation between countries with similar health and economic needs (figure 5). In comparing the two least developed countries with the highest mortality burden, Ethiopia received about $800 million over the study period and DR Congo received less than $400 million. In Angola, $18 was received per death, the lowest of all countries, in contrast with $1037 per death in Liberia, $1183 per death in Haiti, and $1389 per death in Timor-Leste. Nine of the least developed countries received less than $100 per newborn death and stillbirth per year; of these, eight are in Africa (Angola, Burundi, Chad, Congo [Brazzaville], Madagascar, Mauritania, Niger, and Sudan); the other is Yemen. Other high-burden but middle-income countries (eg, India) receive more aid per newborn death and stillbirth than the least developed countries despite having a larger domestic fiscal space for contributions (appendix 2 p 31).

## Discussion

Aid for reproductive, maternal, newborn, and child health increased consistently over the 2002–15 period but has stagnated over 2015–19, with a value of $1·6 billion estimated for 2019 that mentions newborns, despite the increase in policy commitments to newborn survival and stillbirth prevention. Aid mentioning newborns represented 8–11% of aid for reproductive, maternal, newborn, and child health consistently in 2009–19. Funding that mentioned stillbirths was valued at just $44·4 million across the 2002–19 period and stillbirths were not mentioned in the CRS before 2009. Only two donors provided 62% of the aid mentioning stillbirths. Ten donors provided 87% of the aid ($13·7 billion) mentioning newborns and stillbirths over the 2002–19 period. Most recipients were African and Asian countries, and the distribution of aid was broadly aligned with the burden of newborn deaths and stillbirths, but targeting was inconsistent. The most striking finding from our analyses is that stillbirths remain almost completely unmentioned in aid disbursements. Despite a global burden of more than 2 million deaths annually,^23^ terms related to stillbirth occurred in only 46 out of a total of 3·7 million records identified from the CRS.

One strength of our study is that it provides consistent trends about external financing mentioning newborns and stillbirths over almost two decades. Four main approaches to aid tracking in reproductive, maternal, newborn, and child health have been applied and recently assessed, offering differing values to decision makers.^21^ We chose Muskoka2 to provide specific project descriptions to allow for more targeted systematic searching within the ODA portfolio and more granularity within the reproductive, maternal, newborn, and child health continuum for focused terminology and interventions to hold donors accountable for commitments to vulnerable groups.^16^, ^17^, ^20^, ^24^

There are also limitations inherent to the dataset: disbursements are self-reported to the CRS by donors and not all donors are represented. Although many non-OECD countries report disbursements to the CRS, China and Brazil are notably absent, while the Global Financing Facility, which focuses on newborns and stillbirths, does not report separately from the World Bank. Beyond the CRS, AidData has attempted to overcome the challenges of exclusion of emerging donors that might not participate in global reporting systems such as the CRS. Although AidData's Global Chinese Development Finance Dataset does provide brief descriptions, these are primarily drawn from media reports and have an insufficient level of detail; a quick search of the latest available dataset (including years 2000–17) covering overlapping conceptual areas with this analysis uncovered no mentions of “stillbirth”, “newborn”, “neonate”, or “MNCH”. The CRS database gives a description of the purpose of disbursements, and some donors provide less detail than others. The analytical methods used are robust but subject to human error given the large number of records, which we have tried to counteract through double and triple coding a portion of the records. Broadly, interventions on the reproductive, maternal, newborn, and child health continuum are likely to benefit multiple population groups. Our classification of disbursements targeting stillbirth prevention might have underestimated the funding for stillbirth, since funding for maternal health to address some causes of stillbirth (eg, congenital syphilis and malaria in pregnancy) might not mention stillbirth. We view this as a missed opportunity to quantify the impact of aid on a broader set of beneficiaries. Especially for upper-middle-income countries, domestic funds are a major component of health financing, but are more complex to track and not included in these analyses.

Funding for newborns was minimal before 2005, when newborn health emerged on the global agenda.^25^ The *Lancet* Every Newborn Series, published in 2014, described the increases in the previous decade, highlighting missed opportunities.^11^ Analyses by Pitt and colleagues^17^ of aid funding from 2003 to 2013 showed increasing trends in aid mentioning newborns, although stillbirths were rarely mentioned in that period. Our analysis does not show sustained increases in aid mentioning newborns, which aligns with previous findings showing inconsistent aid financing more widely.^16^ Consistent with the 2003–13 analysis by Pitt and colleagues,^17^ we show that funding mentioning stillbirths still remains almost absent among global aid priorities.^26^ This is a reflection on the ongoing lack of visibility of stillbirths, which go unmentioned even when it would be a gain for donors to mention them.^27^, ^28^ Despite major global events, including the global financial crisis in 2007–08 and the launch of the SDGs in 2015 that were predicted to lead to changes in aid allocation and disbursement, we did not observe any such changes in our dataset.

Efficiency of aid for newborns and stillbirths can be improved. Better alignment with the mortality burden could provide greater value for money, enhancing equity and recognising the effect that such deaths have on families and especially women.^13^, ^29^ Our analysis shows that only around 10% of aid for maternal, newborn, and child health mentions newborn deaths and stillbirths globally, and this mismatch is mirrored at the national level, where we see a misalignment between the burden of stillbirths and neonatal mortality and the amount of aid received in UN-designated least developed countries (noting that a country can be considered least developed while not being a low-income country [eg, Angola], which might influence priority for aid). Low levels of funding to least developed countries (as defined by the UN) might reflect efficiency arguments; although such countries have high needs, they might not have the absorptive capacity to make efficient use of such aid. It is difficult to know whether such arguments explain the lower levels of funding to DR Congo, Sudan, Niger, Chad, Guinea, and Mauritania compared with their needs, or whether this is more related to the lack of political priority accorded to these countries. Further efficiencies might be sought by better leveraging funds across the rmnch continuum, including early intervention through pregnancy and childbirth interventions, to support newborn survival and stillbirth prevention.^18^, ^30^ Finally, the global push for value-for-money investments from external financiers addresses economy, efficiency, effectiveness, and cost-effectiveness as general concepts, yet priority setting and decision making might be as much politically driven as data driven, linked to historical allocations and connections, with “neither performance nor impact…a major criterion for allocating resources” in multilateral organisations working in global health.^31^

From these analyses, we draw two main conclusions. First, to maximise impact and promote equity, aid allocation and disbursement must be better aligned with need. Specifically in this context, donors should increase aid focused on preventing stillbirths and improving newborn survival to better align with the related mortality burden of 4·4 million deaths in 2023. Such changes in targeting of aid require ongoing analysis of aid flows to promote accountability including use of data by society and parent groups.. Second, analyses of the political economy of health financing to examine the processes involved and power interests are crucial to inform accountability and action. Our results suggest that aid funding allocations are presently being driven by factors other than the large numbers of deaths, national priorities, or the potential for lives saved.

## Data sharing

The CRS database is publicly available online. Other data are stored in the London School of Hygiene & Tropical Medicine repository and available upon reasonable request via email to the corresponding author.

## Declaration of interests

We declare no competing interests.

## Supplementary Materials

French translation of the abstract
