# Supplementary material for: Donor aid mentioning newborns and stillbirths, 2002–19: an analysis of levels, trends, and equity
Source: Lancet Glob Health. 2023 Oct 17;11(11):e1785–93. doi: 10.1016/S2214-109X(23)00378-9 (PMC10603612; doi:10.1016/S2214-109X(23)00378-9)
Supplement: French translation of the abstract [file mmc1.pdf]

THE LANCET
Global Health

Supplementary appendix 1

This translation in French was submitted by the authors and we reproduce it as supplied. It has not been peer reviewed. *The Lancet*'s editorial processes have only been applied to the original in English, which should serve as reference for this manuscript.

Cette traduction en français a été proposée par les auteurs et nous l'avons reproduite telle quelle. Elle n'a pas été examinée par des pairs. Les processus éditoriaux du *Lancet* n'ont été appliqués qu'à l'original en anglais et c'est cette version qui doit servir de référence pour ce manuscrit.

Supplement to: Kumar MB, Bath D, Binyaruka P, Novignon J, Lawn JE, Catherine Pitt C. Donor aid mentioning newborns and stillbirths, 2002–19: an analysis of levels, trends, and equity. *Lancet Glob Health* 2023; **11:** e1785–93.

Contexte

L'aide mondiale à la santé reproductive, maternelle, néonatale et infantile a stagné ces dernières années, et l'aide mentionnant les nouveau-nés ou les mortinaissances représentait jusqu'à présent une très faible proportion de l'aide à la santé reproductive, maternelle, néonatale et infantile. Des objectifs de survie néonatale ont été fixés par 78 pays et des objectifs de prévention de la mortinatalité ont été fixés par 30 pays, afin de lutter contre les 4.4 millions de décès de nouveau-nés et de mortinaissances dans le monde. Nous avons cherché à produire de nouvelles estimations des niveaux actuels et des tendances de l'aide mentionnant les nouveau-nés et les mortinaissances sur la période 2002-19, et à évaluer si le montant de l'aide versée est en accord avec le fardeau de la mortalité qui y est associé.

Méthodes

Pour cette analyse, nous avons procédé à un examen manuel et à un codage de la base de données du Système de notification des pays créanciers de l'Organisation de coopération et de développement économiques (OCDE) de 2002 à 2019 en utilisant des termes de recherche clés pour l'aide mentionnant les nouveau-nés et les mort-nés. Nous avons comparé ces résultats avec les estimations de l'aide à la santé reproductive, maternelle, néonatale et infantile pour la période 2002-19 basées sur la méthode Muskoka2. Les résultats sont présentés en dollars américains de 2019 selon les déflateurs du Comité d'aide au développement de l'OCDE, qui tiennent compte des variations des taux de change et de l'inflation dans les pays donateurs.

Résultats

Nous avons identifié 21 957 dossiers uniques pour la période 2002-19. L'aide mentionnant les nouveau-nés et les mort-nés représentait environ 10 % (1.6 milliards de dollars) du financement global de la santé reproductive, maternelle, néonatale et infantile en 2019 (15.9 milliards de dollars), avec une légère diminution de la valeur entre 2015 et 2019. 1284 (6 %) des 21 957 dossiers et 3.4 % (535 millions de dollars) de leur valeur totale mentionnaient une aide axée uniquement sur la santé des nouveau-nés. Dix donateurs ont contribué à 87 % (13.7 milliards de dollars) de la valeur totale de l'aide mentionnant les nouveau-nés et les mort-nés au cours de la période 2002-19. L'aide mentionnant les nouveau-nés et les mort-nés a été allouée de manière inéquitable dans les pays les moins avancés (tels que définis par les Nations unies), allant de 18 dollars par décès en Angola à 1389 dollars par décès au Timor-Oriental. Les mortinaissances n'ont été mentionnées dans aucun financement en 2002-2009, et elles n'ont été mentionnées que dans 46 des 21 957 dossiers en 2010-19, ce qui représente 44.4 millions de dollars d'aide versée au cours de cette période.

Interprétation

L'aide mentionnant les nouveau-nés et les mort-nés est mal accordée à la charge de mortalité correspondante (représentant 10 % de l'aide à la santé reproductive, maternelle, néonatale et infantile en général, mais environ 50 % de la mortalité des enfants de moins de 5 ans) et entre les pays bénéficiaires (avec des variations substantielles dans le montant de l'aide reçue par décès de nouveau-né et de mort-né entre des pays ayant des besoins économiques et sanitaires similaires). Nos conclusions indiquent que l'aide doit être mieux ciblée sur les populations les plus affectées par la mortalité, afin de créer un plus grand potentiel d'impact.

Financement

John D. and Catherine T. MacArthur Foundation, Bill & Melinda Gates Foundation, ELMA Philanthropies, Children's Investment Fund Foundation UK, Lemelson Foundation, and Ting Tsung and Wei Fong Chao Foundation.
