# Supplementary material for: Donor aid mentioning newborns and stillbirths, 2002–19: an analysis of levels, trends, and equity
Source: Lancet Glob Health. 2023 Oct 17;11(11):e1785–93. doi: 10.1016/S2214-109X(23)00378-9 (PMC10603612; doi:10.1016/S2214-109X(23)00378-9)
Supplement: Supplementary appendix 2 [file mmc2.pdf]

THE LANCET
Global Health

Supplementary appendix 2

This appendix formed part of the original submission and has been peer reviewed. We post it as supplied by the authors.

Supplement to: Kumar MB, Bath D, Binyaruka P, Novignon J, Lawn JE, Catherine Pitt C. Donor aid mentioning newborns and stillbirths, 2002–19: an analysis of levels, trends, and equity. *Lancet Glob Health* 2023; **11:** e1785–93.

Table of Contents

Additional information on Methods 2

S1: Search terms utilized 2

S2: Coding strategy and detailed methodological notes 6

Additional Results 7

S3: Trends in global aid mentioning newborns for donors reporting in different years 7

S4: Proportion of global aid to RMNCH, MNCH and MNH mentioning newborns and stillbirths, 2002-19 8

S5: All records mentioning stillbirth across entire study period 2002-19 (n=46), sorted by decreasing value of disbursement 9

S6: Absolute value of global aid mentioning newborns and stillbirths, by CRS purpose code, 2002-19 28

S7: Global aid mentioning newborns and stillbirths by donor, 2002-19 29

S8: Value of aid by donor / by year for top donors (million 2019USD) 30

S9. Absolute value of global aid mentioning newborns and stillbirths by recipient in the context of Muskoka2 MNH estimates, 2002-19 31

S10. Number of neonatal deaths and stillbirths and amount of aid received in Least Developed Countries, 2002-2019 32

Additional information on Methods

S1: Search terms utilized

Listed below are (a) the search terms included in the analysis ('Included search terms') and (b) the search terms considered but excluded from the analysis ('Excluded search terms'). Where a search term includes a space before/after/between words, this is shown below as an underscore ("_"). Where a search involved two non-adjacent terms, these are separated by "%".

* indicates the top 3 search terms by total number of records found from 2002-2019:

newborn (13,986 records; 50.4% of all records found across all search terms),
neonat (5,031; 18.1%), and
mnch (4,412; 15.9%).

Included search terms

newborn *
mnch *
nouveau-ne
smn
smne
neonat *
tt_
_tt
breastfe
allaite
_allaite
mnh
postnat
perinat
post-nat
prematur
preterm
tpi
nouveau_ne
breast_fe
fetal
imnci
_fetal
kangaro
iptp
recien_nacido
stillb
faible_poids
fetus
_fetus
birth_weight
_lactanc
lactanc
post_nat

asphyxi
feto
syphilis
bajo_peso
malaria_in_pregnan
umbilic
_cordon
jaundice
neo-nat
skin-to-skin
_feto
_foetal
antenatal_steroid
birthweight
breast-fe
cord_care
foetal
foetus
nace%muerto
paludi%_enceint
posnata
sifilis
toxoid
traitement_preventif_intermittent
continuous_positive_airway_pressure

Excluded search terms

Terms were excluded if they found no records in both the previous analysis (Pitt C, et al. *BMJ Global Health* 2017) and a new search of CRS disbursements for 2019. For a term was added since the previous analysis, it was excluded if the term found no records in the 2019 CRS disbursements.

amamant
borstvoed
cangu
intermittent_preventative_treatment%pregnan
malaria_%_embaraz
malaria%_schwanger
miscarriage
mort-ne
neugeboren
pos_nata
pos-nata
prematuur
recem-nascido
aborto_espont
aborto_spont
allatta
anatoxin

asfissia
avortement_spont
baixo_peso
canguro
canguru
cpap
doodgebor
doogebor
fehlgeburt
foetaal
fruhes_stillen
fruhgeburt
geboorte_gewicht
geboortegewicht
geburtsgewicht
gelbsucht
hautkontakt
ictericia
ikterus
intermittent_presumptive_treatment
itterizia
jauniss
kangourou
kanguru
laktat
lattazione
lues_connata
malaria_%_gravid
malaria_in%zwanger
miskraam
mort_ne
mortinaissance
mortinatalidad
mortinato
nabelinfektion
nabelpflege
nacido_muerto
nasce%morto
nascido_muito_cedo
natimorto
nato_mort
navelstreng
neonaat
ombelical
ombilical
paludi%_embaraz
paludi%_gravid
pasgeboren

Pcimni
peau%peau
pele_a_pele
pelle_a_pelle
peso_al_nacer
peso_de_nacimiento
piel_con_piel
poids_a_la_naissance
poids_de_naissance
recem_nascideo
sifilide
skin_to_skin
sottopeso
spontane_afbreking
tetanique
tetanusimpfung
totgeb
traitement_presomptif_intermittent
tratamento_intermitente_preventivo
tratamiento_preventivo_intermitente%embaraz
umbigo
unreif
untergewicht
vroeg_geboorte
wochenbett

S2: Coding strategy and detailed methodological notes

Appendix S2 expands on the detail provided in the methods section to clarify how differences in coding were checked and resolved.
Double coding was done for the following categories of records:

1) all records flagged by the main coder (DB) as uncertain or for review
2) all unique titles with a value over $7 million
3) a randomly selected 5% sample.

DB and MBK coded a subset blindly and then met to review. Where the coding was the same, we discussed what coding rules were evolving and continued with a shared understanding. Where the double coding differed or uncertainty persisted, blind triple coding was conducted by CP and discussion went on to reach coding rules.

Coding was done in an iterative process where both DB and MBK would do an overlapping portion of assigned records, compare to discuss and reach a shared understanding, and continuation. Rules reached as a consensus view for inclusion/exclusion are described in the table below.

**Rule**	**Exceptions**
Exclude records with references to PMTCT	Mentions additional newborn activities other than PMTCT
Exclude HIV integrated into MNCH	Mentions additional newborn activities other than HIV
Where there is aggregated term for MNH or MNCH but information only on neonatal-specific activities, code as focused funding	
(Preventing) preterm birth – code as focused funding	Eclampsia/pre-eclampsia are included
Basic animal research targeted towards improving fetal/newborn health or drug candidates – code as includes	
If outcomes are newborn, code as focused funding (even if intervention is done 'to' mother)	
If long description is consistent but more narrow/specific than short description, use the more narrow as the basis of coding	Threshold of exclude / include looser than threshold between focused / include, so better to include anything uncertain but 'focused' interventions are rare given the nature of the continuum of care
Nouakchott records pulled up many false tt matches intended to be for tetanus toxoid; check for other key terms, otherwise exclude	Unless mention newborn or stillbirth terms other than 'tt'
Neonatal morbidity has lifelong potential disability (eg retinopathy of prematurity) – code as focused funding	If surgery / interventions are for non-newborn
Birth registration - coded as 'includes' newborn	
Malaria or syphilis interventions - exclude	only include if 'malaria in pregnancy' or congenital syphilis

Additional Results

S3: Trends in global aid mentioning newborns for donors reporting in different years

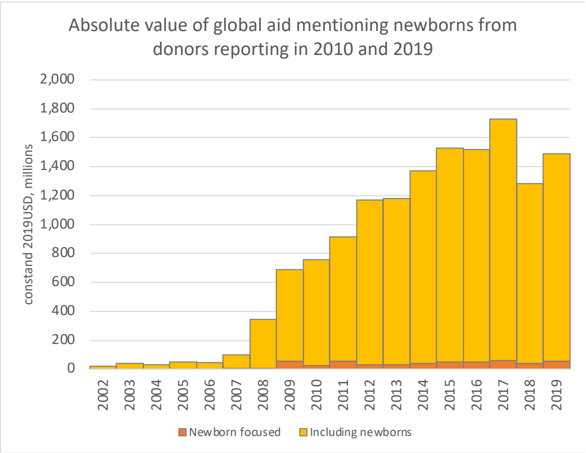

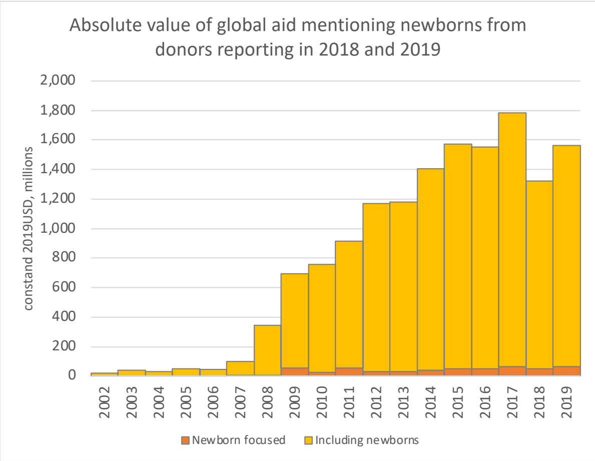

S4: Proportion of global aid to RMNCH, MNCH and MNH mentioning newborns and stillbirths, 2002-19

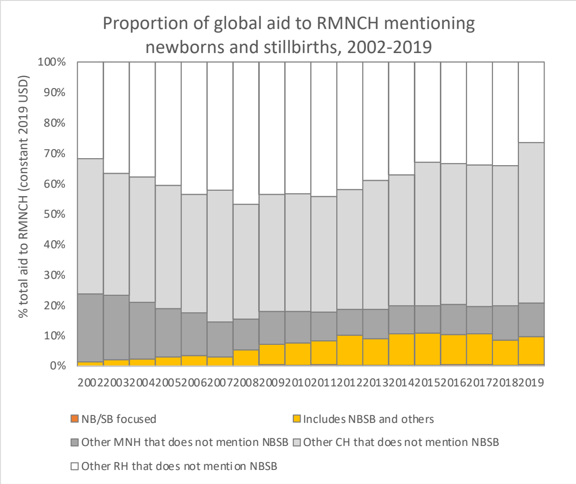

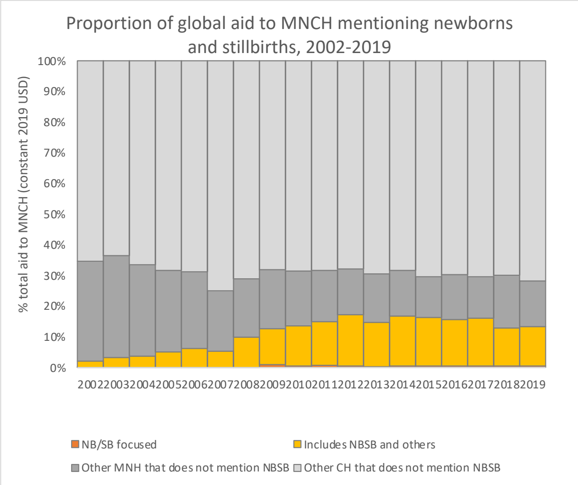

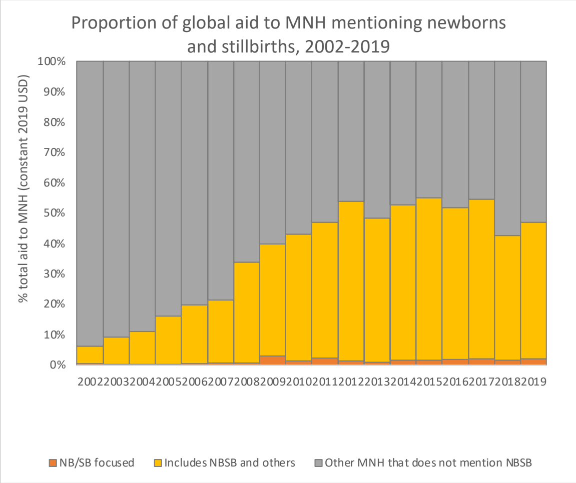

S5: All records mentioning stillbirth across entire study period 2002-19 (n=46), sorted by decreasing value of disbursement

Year	Donor	Recipient	Purpose Code	Flow Type	Disbursement (million USD)	Project Title	Long Description
2013	Bill & Melinda Gates Foundation	Bilateral, unspecified	Medical research	Private Development Finance	8.20983	Seattle Childrens Hospital Foundation	to generate scientific discoveries that will be made readily available to the global community to facilitate more rapid development of equitable and context-relevant interventions for prevention of preterm birth and stillbirth
2011	Bill & Melinda Gates Foundation	Bilateral, unspecified	Medical research	Private Development Finance	6.90104	Seattle Childrens Hospital Foundation	to generate scientific discoveries that will be made readily available to the global community to facilitate more rapid development of equitable and context-relevant interventions for prevention of preterm birth and stillbirth
2019	Wellcome Trust	South of Sahara, regional	Reproductive health care	Private Development Finance	5.24817	PRECISE-DYAD: linking maternal and infant health trajectories in sub-Saharan Africa	Programme: Collaborative Award in Science. Description: PRECISE-DYAD will extend the ongoing PRECISE network study [PRECISEnetwork.org] of 6,000 pregnant women and their infants (dyads); it will provide a greater epidemiological and mechanistic understanding of health and disease pathways in the sub-Saharan African context (The Gambia and Kenya), following uncomplicated pregnancies and those complicated by pregnancy hypertension, fetal growth restriction, stillbirth, and/or preterm birth. For women, we aim to determine if, and how, these pregnancy events identify those more likely to avoid complications in subsequent pregnancies, mental ill-health, or early onset of cardiometabolic diseases. For their children, we will assess the impact of these pregnancy disorders on their physical, mental, and neurodevelopmental health to 2-3 years of age. We will explore the consequences for maternal health trajectories of caring for children with moderate-to-severe neurodevelopmental delay resulting from complicated pregnancies, and will interrogate interactions with social determinants of health, including environmental exposures on maternal and infant health. We will develop a rich database and a large sample biorepository for future studies. Thereby, PRECISE-DYAD aims to achieve an overarching understanding of the factors that contribute to optimal maternal and child health within these contexts. Ultimately, the goal is to design interventions to optimise health of mothers and their children.

Year	Donor	Recipient	Purpose Code	Flow Type	Disbursement (million USD)	Project Title	Long Description
2015	Bill & Melinda Gates Foundation	Bilateral, unspecified	Medical research	Private Development Finance	3.97489	Seattle Childrens Hospital Foundation	to generate scientific discoveries that will be made readily available to the global community to facilitate more rapid development of equitable and context-relevant interventions for prevention of preterm birth and stillbirth
2018	Children's Investment Fund Foundation	Bilateral, unspecified	Reproductive health care	Private Development Finance	3.49274	London School of Hygiene and Tropical Medicine (LSHTM): Transforming Measurement for Every Newborn Phase 2	Programme: Childhood. Description: Transforming Measurement for Every Newborn Phase 2 - Transforming Measurement for Every Newborn Phase 2. To enable evidence-based decision making and accelerate progress towards the goal of dramatically reducing the number of newborn deaths and stillbirths by 2030..
2010	Switzerland	Tanzania	Reproductive health care	ODA Grants	2.20466	Emergency Obstetric Care (EmOC)	The progress of the health outcomes in Tanzania is impressive within the last decade which is reflected in the reduction of the child mortality by 40% between 1999 and 2008. Nevertheless the reduction of newborn and child mortality remains a high priority for the Government - beside the reduction of persistently high maternal mortality. Both are reflected in the MDG 4 and 5, and in Tanzania under the National Strategy for Growth and Reduction of Poverty and the Health Sector Strategic Plan. Tanzania is among the ten countries in Africa with the highest number of maternal and neo-natal deaths. Tanzania Maternal Mortality Ratio has remained high for the past 10 years and is currently estimated to be at 578 per 100,000 live births (2005). Similarly, Neo-natal mortality rate has not decreased over the years and is at approximately 32 per 1000 live births (2002 / 2005). Each year 8,500 women die from pregnancy related causes and childbirth in Tanzania. In addition, 250,000 women every year suffer disabling conditions as a result of pregnancy and childbirth. The risk of maternal death is 50 times higher in Tanzania compared to developed world. Only 46% of births are attended by a skilled health worker (2010) and there are 30% fewer doctors than required. Each year at least 51,000 Tanzanian newborns die, and 43,000 babies are stillborn. Up to two-thirds, or 34,000 newborn lives could be saved if essential care reached mothers and babies. Maternal and new born deaths are caused by factors attributable to pregnancy, childbirth and poor quality of health services including lack of essential equipment. In order to improve maternal, newborn and child care, Tanzania has launched the National Road Map Strategic Plan to Accelerate Reduction of Maternal,

Year	Donor	Recipient	Purpose Code	Flow Type	Disbursement (million USD)	Project Title	Long Description
							Newborn and Child Deaths in Tanzania/One Plan (2008 - 2015) in May 2008. The Ministry of Health and Social Welfare requested the World Bank to assist in funding a component of this plan, and to allocate part of their USD 40 million loan for the direct purchase of Emergency Obstetric and Newborn Care (EmONC) Equipments. The urgent need for EmONC instruments in public health facilities became evident in the situation analysis which was done in 2006 .The World Bank agreed to the request. SDC contribution will allow more hospitals and health centres to be equipped. Unicef and One UN system will provide technical assistance and capacity building which is required for a proper use of the instruments. The overall objective of the project is to contribute to providing higher quality services and safer deliveries for pregnant women, as well as improved care for the neonates in Tanzania. The impact hypothesis is that this contributes to a reduction of maternal and neonatal mortality and morbidity. The Swiss contribution will cover EmONC instruments for approximately 15 district hospitals and 30 health centres. The beneficiaries are the women and newborns; however the staffs in the health facilities are the direct recipien
2018	Sweden	Zambia	Basic nutrition	ODA Grants	2.08276	UNICEF Micronutrient and Food Consumption Survey - Micronutrient and Food Consumption Survey	UNICEF will support the government of Zambia through the National Food and Nutrition Commission of Zambia to implement a National Micronutrient and Food Consumption Survey to inform strategies and policies. The objectives of the project are to describe the micronutrient intakes patterns and to measure the effective coverage of current nutrition interventions amongst targeted populations in Zambia, and their effect on the micronutrient status. A large proportion of children in Zambia are estimated to suffer from stunted growth, cognitive delays, weakened immunity and disease because of micronutrient deficiencies. For pregnant women, the lack of essential vitamins and minerals can be catastrophic, increasing the risk of low birth weight, birth defects, stillbirth, and maternal death. Programmes to prevent and treat micronutrient deficiencies in children and at-risk population are have been implemented over years but there are considerable knowledge gaps on the coverage and effects of those interventions. This survey will provide information on the effectiveness and coverage of interventions to reduce micronutrient deficiencies and inform the development of improved micronutrient control strategies, and national dietary guidelines. The survey will also ensure accurate information for contribution to regional and global reports.

Year	Donor	Recipient	Purpose Code	Flow Type	Disbursement (million USD)	Project Title	Long Description
2017	Bill & Melinda Gates Foundation	Bilateral, unspecified	Medical research	Private Development Finance	1.64395	Global Alliance to Prevent Prematurity and Stillbirth	to generate scientific discoveries that will be made readily available to the global community to facilitate more rapid development of equitable and context-relevant interventions for prevention of preterm birth and stillbirth
2017	Bill & Melinda Gates Foundation	India	Basic health care	Private Development Finance	1.30244	Indian Council of Medical Research	to support the implementation of a pilot study to establish the feasibility of post-mortem minimally invasive tissue sampling (MITS) among approximately 200 children under five years and stillbirths in a tertiary hospital setting in New Delhi, India
2018	United Kingdom	South of Sahara, regional	Medical research	ODA Grants	1.08489	NIHR Global Health Research Group on Stillbirth Prevention and Management in Sub-Saharan Africa	A UK and low- and middle-income country (LMIC) partnership that aims to improve the prevention and management of stillbirth
2014	Bill & Melinda Gates Foundation	Nigeria	Malaria control	Private Development Finance	1.06544	JSI Research & Training Institute, Inc.	to demonstrate and document the how-to of at-scale delivery of accessible intermittent preventative treatment of malaria in pregnancy (IPTp) and the expected ancillary benefits of a reduction in the prevalence of low birthweight and in stillbirths
2019	United Kingdom	Bilateral, unspecified	Medical research	ODA Grants	0.890858	NIHR Global Health Research Group on Stillbirth Prevention and Management in Sub-Saharan Africa	A UK and low- and middle-income country (LMIC) partnership that aims to improve the prevention and management of stillbirth.
2017	Children's Investment Fund Foundation	Bilateral, unspecified	Reproductive health care	Private Development Finance	0.865796	London School of Hygiene and Tropical Medicine (LSHTM)	Programme: Childhood. Description: Transforming Measurement for Every Newborn Phase 2 - Transforming Measurement for Every Newborn Phase 2. To enable evidence-based decision making and accelerate progress towards the goal of dramatically reducing the number of newborn deaths and stillbirths by 2030.

Year	Donor	Recipient	Purpose Code	Flow Type	Disbursement (million USD)	Project Title	Long Description
2017	Children's Investment Fund Foundation	Bilateral, unspecified	Reproductive health care	Private Development Finance	0.856184	London School of Hygiene and Tropical Medicine (LSHTM)	Programme: Childhood. Description: Transforming Measurement for Every Newborn Phase 2 - Transforming Measurement for Every Newborn Phase 2. To enable evidence-based decision making and accelerate progress towards the goal of dramatically reducing the number of newborn deaths and stillbirths by 2030.
2019	United Kingdom	Bilateral, unspecified	Medical research	ODA Grants	0.721403	NIHR Global Health Research Group on Preterm Birth and Stillbirth (the DIPLOMATIC Collaboration)	A UK and low- and middle-income country (LMIC) partnership that aims to reduce preterm birth and stillbirth and to optimise outcomes for babies born preterm.
2016	Bill & Melinda Gates Foundation	Bilateral, unspecified	Medical research	Private Development Finance	0.642886	Seattle Childrens Hospital Foundation	to generate scientific discoveries that will be made readily available to the global community to facilitate more rapid development of equitable and context-relevant interventions for prevention of preterm birth and stillbirth
2017	Charity Projects Ltd (Comic Relief)	Ethiopia	Reproductive health care	Private Development Finance	0.464958	Doctors with Africa Cuamm	Primary goal (Comic Relief): Health. Related issues (Comic Relief): Maternal, neonatal, child health. Summary: Each year in Ethiopia, over 100,000 children die during the mothers pregnancy, childbirth, or in the first weeks of life. Many deaths could be prevented if women used maternal health services, if carers knew the danger signs during pregnancy and in newborns, and took action to get help in time, and if babies in severe danger got better care. This project will use tested approaches that complement the national primary health programme. Through groups, women will learn how to keep themselves and their babies safe and healthy. The project will improve the quality of care provided by health workers and outreach workers. This will result in fewer stillbirths and fewer deaths of newborns. Big Lottery Fund funded 91 percent of this grant but the whole amount is attributed to Comic Relief as no double-counting occurs. Full grant commitment: GBP 709.999 thousand.
2012	Australia	Bilateral, unspecified	Basic nutrition	ODA Grants	0.402865	2011-12 Core funding to ICCIDD	Iodine deficiency is the most common cause of preventable mental retardation and brain damage. Serious iodine deficiencies during pregnancy can result in stillbirth, spontaneous abortion, and congenital abnormalities such as cretinism. This initiative provides core funding to International Council for Control of Iodine Deficiency (ICCIDD) to assist developing countries to put in place and maintain systems for ensuring sustainable

Year	Donor	Recipient	Purpose Code	Flow Type	Disbursement (million USD)	Project Title	Long Description
							optimal iodine nutrition. The total value of this multi-country initiative is $500,000 for the 2011-12 financial year.
2019	Charity Projects Ltd (Comic Relief)	Ethiopia	Reproductive health care	Private Development Finance	0.327099	Doctors with Africa Cuamm	Primary goal (Comic Relief): Health. Related issues (Comic Relief): Maternal, neonatal, child health. Summary: Each year in Ethiopia, over 100,000 children die during the mothers pregnancy, childbirth, or in the first weeks of life. Many deaths could be prevented if women used maternal health services, if carers knew the danger signs during pregnancy and in newborns, and took action to get help in time, and if babies in severe danger got better care. This project will use tested approaches that complement the national primary health programme. Through groups, women will learn how to keep themselves and their babies safe and healthy. The project will improve the quality of care provided by health workers and outreach workers. This will result in fewer stillbirths and fewer deaths of newborns. Big Lottery Fund funded 91 percent of this grant but the whole amount is attributed to Comic Relief as no double-counting occurs. Full grant commitment: GBP 709.999 thousand.
2018	Bill & Melinda Gates Foundation	Bilateral, unspecified	Medical research	Private Development Finance	0.28	Grand Challenges in Global Health: Healthy Birth Initiative	to generate scientific discoveries that will be made readily available to the global community to facilitate more rapid development of equitable and context-relevant interventions for prevention of preterm birth and stillbirth
2010	Bill & Melinda Gates Foundation	Bilateral, unspecified	Reproductive health care	Private Development Finance	0.235431	Mater Medical Research Institute	to produce a series of papers for The Lancet placing stillbirths as a priority within the context of maternal and child survival to promote action and guide priority-setting for programs and research

Year	Donor	Recipient	Purpose Code	Flow Type	Disbursement (million USD)	Project Title	Long Description
2018	Wellcome Trust	Bilateral, unspecified	Infectious disease control	Private Development Finance	0.213419	Structural Characterisation of the Orthobunyaviral Surface Glycoproteins	Programme: PhD Studentship (Basic). Description: Orthobunyaviruses present medical and economical threats as they cause haemorrhagic fever and encephalitis in human, and abortion and stillbirth in livestock. Currently, little is known about how orthobunyaviruses infect their hosts and how they achieve cross-species transmission from anthropods to humans. My DPhil research focuses on structurally characterising the glycoproteins utilised by the orthobunyaviruses for infection and screening for neutralising antibodies. Using X-ray crystallography and electron microscopy techniques, I aim to contribute to a more complete understanding of orthobunyavirus-host cell attachment, intracellular trafficking, and membrane fusion. Ultimately, knowledge of host-cell entry mechanism will aid the development of vaccines and inhibitive peptides. . Note: commitment amount is approximate.
2015	Bill & Melinda Gates Foundation	Nigeria	Malaria control	Private Development Finance	0.210475	JSI Research & Training Institute, Inc.	to demonstrate and document the how-to of at-scale delivery of accessible intermittent preventative treatment of malaria in pregnancy (IPTp) and the expected ancillary benefits of a reduction in the prevalence of low birthweight and in stillbirths
2018	Bill & Melinda Gates Foundation	India	Basic health care	Private Development Finance	0.17826	A Pilot Study to Determine Causes of Death in Under-Five Children in a Tertiary Hospital in India using the MITS Technique	to support the implementation of a pilot study to establish the feasibility of post-mortem minimally invasive tissue sampling (MITS) among approximately 200 children under five years and stillbirths in a tertiary hospital setting in New Delhi, India
2018	Charity Projects Ltd (Comic Relief)	Ethiopia	Reproductive health care	Private Development Finance	0.134737	Doctors with Africa Cuamm	Summary: Each year in Ethiopia, over 100,000 children die during the mothers pregnancy, childbirth, or in the first weeks of life. Many deaths could be prevented if women used maternal health services, if carers knew the danger signs during pregnancy and in newborns, and took action to get help in time, and if babies in severe danger got better care. This project will use tested approaches that complement the national primary health programme. Through groups, women will learn how to keep themselves and their babies safe and healthy. The project will improve the quality of care provided by health workers and outreach workers. This will result in fewer stillbirths and fewer deaths of newborns. Big Lottery Fund funded 91 percent

Year	Donor	Recipient	Purpose Code	Flow Type	Disbursement (million USD)	Project Title	Long Description
							of this grant but the whole amount is attributed to Comic Relief as no double-counting occurs. Full grant commitment: GBP 709.999 thousand.
2019	Sweden	Bilateral, unspecified	Medical research	ODA Grants	0.134308	Scaling-up Helping Babies Breathe Quality Improvement Cycle (HBB-QIC) at district level in Nepal	To further accelerate the rate of reduction of early neonatal mortality and intrapartum stillbirths a simplified neonatal resuscitation protocol: Helping Babies Breathe (HBB) has been developed, which has been shown to reduce intra-partum deaths by about 50%. There is however a need to evaluate different implementation strategies that promote retention of resuscitation skills and facilitate translation of knowledge into clinical practice, and to scale-up such implementation strategies. We have previously successfully completed an implementation trial of HBB at a tertiary hospital in Kathmandu, Nepal. The HBB protocol was complemented by a Quality Improvement Cycle strategy (QIC) and displayed good retention of skills and significant changes in clinical practice. We now propose a scale-up study at district level of the HBB-QIC concept. The research project till apply a stepped-wedge cluster-randomized controlled design at eight (8) district hospital in different regions of Nepal. To ensure good health and survival for all is at the core of the development research agenda and this project addresses a neglected area with great potential for improvement. With a successful scale-up strategy the HBB-QIC concept can potentially have far-reaching consequences and make a huge contribution to the efforts to reduce the burden of intrapartum deaths.
2017	Sweden	Bilateral, unspecified	Fishery research	ODA Grants	0.129372	Testing the potential of microalga-bacteria consortium to manage pathogens in cultured shrimps	To further accelerate the rate of reduction of early neonatal mortality and intrapartum stillbirths a simplified neonatal resuscitation protocol: Helping Babies Breathe (HBB) has been developed, which has been shown to reduce intra-partum deaths by about 50%. There is however a need to evaluate different implementation strategies that promote retention of resuscitation skills and facilitate translation of knowledge into clinical practice, and to scale-up such implementation strategies. We have previously successfully completed an implementation trial of HBB at a tertiary hospital in Kathmandu, Nepal. The HBB protocol was complemented by a Quality Improvement Cycle strategy (QIC) and displayed good retention of skills and significant changes in clinical practice. We now propose a scale-up study at

Year	Donor	Recipient	Purpose Code	Flow Type	Disbursement (million USD)	Project Title	Long Description
							district level of the HBB-QIC concept. The research project till apply a stepped-wedge cluster-randomized controlled design at eight (8) district hospital in different regions of Nepal. To ensure good health and survival for all is at the core of the development research agenda and this project addresses a neglected area with great potential for improvement. With a successful scale-up strategy the HBB-QIC concept can potentially have far-reaching consequences and make a huge contribution to the efforts to reduce the burden of intrapartum deaths.
2019	Bill & Melinda Gates Foundation	Bilateral, unspecified	Infectious disease control	Private Development Finance	0.127724	Exploring the potential use of Next Generation Sequencing for Molecular C	to reduce neonatal deaths and stillbirths from infectious diseases by using next generation sequencing and IDseq software on blood samples to identify causative pathogens and help develop more effective diagnostics and treatments
2019	Bill & Melinda Gates Foundation	Ghana	Basic nutrition	Private Development Finance	0.0982287	Adverse Outcome in Pregnancy Trial (AdOPT) Africa: Planning Grant	to generate evidence for a group of antenatal nutrient interventions in preventing adverse birth outcomes including preterm birth, preeclampsia and stillbirth
2018	Sweden	Bilateral, unspecified	Medical research	ODA Grants	0.0805487	Scaling-up Helping Babies Breathe Quality Improvement Cycle (HBB-QIC) at district level in Nepal	To further accelerate the rate of reduction of early neonatal mortality and intrapartum stillbirths a simplified neonatal resuscitation protocol: Helping Babies Breathe (HBB) has been developed, which has been shown to reduce intra-partum deaths by about 50%. There is however a need to evaluate different implementation strategies that promote retention of resuscitation skills and facilitate translation of knowledge into clinical practice, and to scale-up such implementation strategies. We have previously successfully completed an implementation trial of HBB at a tertiary hospital in Kathmandu, Nepal. The HBB protocol was complemented by a Quality Improvement Cycle strategy (QIC) and displayed good retention of skills and significant changes in clinical practice. We now propose a scale-up study at district level of the HBB-QIC concept. The research project till apply a stepped-wedge cluster-randomized controlled design at eight (8) district

Year	Donor	Recipient	Purpose Code	Flow Type	Disbursement (million USD)	Project Title	Long Description
							hospital in different regions of Nepal. To ensure good health and survival for all is at the core of the development research agenda and this project addresses a neglected area with great potential for improvement. With a successful scale-up strategy the HBB-QIC concept can potentially have far-reaching consequences and make a huge contribution to the efforts to reduce the burden of intrapartum deaths.
2017	United Kingdom	South of Sahara, regional	Medical research	ODA Grants	0.0679604	NIHR Global Health Research Group on Stillbirth Prevention and Management in Sub-Saharan Africa	A UK and low- and middle-income country (LMIC) partnership that aims to improve the prevention and management of stillbirth
2018	United Kingdom	South of Sahara, regional	Medical research	ODA Grants	0.0666933	NIHR Global Health Research Group on Preterm Birth and Stillbirth (the DIPLOMATIC Collaboration)	A UK and low- and middle-income country (LMIC) partnership that aims to reduce preterm birth and stillbirth and to optimise outcomes for babies born preterm.
2015	Sweden	Nepal	Reproductive health care	ODA Grants	0.0320742	SCALING-UP INTERVENTIONS FOR QUALITY OF CARE IMPROVEMENTS IN THE FIELD OF MATERNAL AND CHILD HEALTH CARE IN NEPAL	This project will be carried out in Nepal in collaboration with Nepalese partners. The objective is to develop capacity and collaboration on health care system research in and between Nepal and Sweden. This will be done through a series of intervention studies focusing on improvements in quality of care in the arena of maternal and child health care. This will be achieved through collaboration between UNICEF, Uppsala University (UU) and Patan Academy of Health Sciences (PAHS) in Kathmandu. UU and PAHS have since 2012 collaborated on a hospital-based intervention trial implementing a simplified neonatal resuscitation protocol (Helping Babies Breath, HBB) at a tertiary level delivery hospital in Kathmandu. The study period for the trial was completed in September 2013. Preliminary results indicate a 53% reduction of intra-partum stillbirths and considerable improvements in the quality of delivery care. Ministry of Health and Population (MoHP), which have commissioned UNICEF to scale up the HBB intervention at 40 district hospitals. We plan to design a series of research studies investigating

Year	Donor	Recipient	Purpose Code	Flow Type	Disbursement (million USD)	Project Title	Long Description
							different approaches on the implementation of QoC enhancing interventions. Considering the programmatic approach in the call for applications we intend to integrate a number of research trials over the program period, thus being able to use synergies and develop a critical mass for the Swedish-Nepalese research collaboration.
2016	Sweden	Nepal	Reproductive health care	ODA Grants	0.028946	Scaling-up interventions for quality of care improvements in the field of maternal and child health care in Nepal	This project will be carried out in Nepal in collaboration with Nepalese partners. The objective is to develop capacity and collaboration on health care system research in and between Nepal and Sweden. This will be done through a series of intervention studies focusing on improvements in quality of care in the arena of maternal and child health care. This will be achieved through collaboration between UNICEF, Uppsala University (UU) and Patan Academy of Health Sciences (PAHS) in Kathmandu. UU and PAHS have since 2012 collaborated on a hospital-based intervention trial implementing a simplified neonatal resuscitation protocol (Helping Babies Breath, HBB) at a tertiary level delivery hospital in Kathmandu. The study period for the trial was completed in September 2013. Preliminary results indicate a 53% reduction of intra-partum stillbirths and considerable improvements in the quality of delivery care. Ministry of Health and Population (MoHP), which have commissioned UNICEF to scale up the HBB intervention at 40 district hospitals. We plan to design a series of research studies investigating different approaches on the implementation of QoC enhancing interventions. Considering the programmatic approach in the call for applications we intend to integrate a number of research trials over the program period, thus being able to use synergies and develop a critical mass for the Swedish-Nepalese research collaboration.
2017	Sweden	Nepal	Reproductive health care	ODA Grants	0.0289323	Scaling-up interventions for quality of care improvements in the field of maternal and child health care in Nepal	This project will be carried out in Nepal in collaboration with Nepalese partners. The objective is to develop capacity and collaboration on health care system research in and between Nepal and Sweden. This will be done through a series of intervention studies focusing on improvements in quality of care in the arena of maternal and child health care. This will be achieved through collaboration between UNICEF, Uppsala University (UU) and Patan Academy of Health Sciences (PAHS) in Kathmandu. UU and PAHS have since 2012 collaborated on a hospital-based intervention trial implementing a simplified neonatal resuscitation protocol (Helping Babies Breath, HBB) at a tertiary level delivery hospital in Kathmandu. The study period for the trial was completed in September 2013. Preliminary results indicate a 53% reduction of intra-partum stillbirths and considerable improvements in the quality of delivery care. Ministry of Health and Population (MoHP), which

Year	Donor	Recipient	Purpose Code	Flow Type	Disbursement (million USD)	Project Title	Long Description
							have commissioned UNICEF to scale up the HBB intervention at 40 district hospitals. We plan to design a series of research studies investigating different approaches on the implementation of QoC enhancing interventions. Considering the programmatic approach in the call for applications we intend to integrate a number of research trials over the program period, thus being able to use synergies and develop a critical mass for the Swedish-Nepalese research collaboration.
2017	Sweden	Nepal	Agricultural research	ODA Grants	0.0170536	Inducing novel resistance in wheat towards stem rust (Ug99) - for improved food security in East Africa, Middle East and Central Asia	The time before and after birth, the perinatal period, is the time of an annual 300,000 maternal deaths, 3 million stillbirths and 3 million newborn deaths. There is robust evidence that use of facilitated participatory groups is an effective social innovation to prevent a large proportion of these deaths to happen. No large scale-up of this promising innovation has so far taken place. The aim of this project is to adapt, implement, evaluate and communicate a contextually appropriate social innovation, the PeriScope model, for perinatal health in Vietnam and Nepal representing low - high maternal and neonatal mortality. The project targets primary and secondary health system levels and engage stakeholder groups with more than 5,400 key members, health care staff and managers to identify and act on local challenges, quality of care issues and health system obstacles. The project will be based on problem-solving cycles and study its effect on 388,000 births, to our knowledge the largest implementation study in this field. The PeriScope will make health systems more responsive and sustainable, reduce barriers currently preventing the uptake of safe, efficient and equitable perinatal health services. This project will allow for researcher with complimentary skills to embark on previous achievements and develop a strong collaboration. Lessons from this scale-up can be transferred to other settings, enabled by the active participation of the WHO, the UNICEF, and the World Bank.

Year	Donor	Recipient	Purpose Code	Flow Type	Disbursement (million USD)	Project Title	Long Description
2017	Sweden	Viet Nam	Agricultural research	ODA Grants	0.0170536	Inducing novel resistance in wheat towards stem rust (Ug99) - for improved food security in East Africa, Middle East and Central Asia	The time before and after birth, the perinatal period, is the time of an annual 300,000 maternal deaths, 3 million stillbirths and 3 million newborn deaths. There is robust evidence that use of facilitated participatory groups is an effective social innovation to prevent a large proportion of these deaths to happen. No large scale-up of this promising innovation has so far taken place. The aim of this project is to adapt, implement, evaluate and communicate a contextually appropriate social innovation, the PeriScope model, for perinatal health in Vietnam and Nepal representing low - high maternal and neonatal mortality. The project targets primary and secondary health system levels and engage stakeholder groups with more than 5,400 key members, health care staff and managers to identify and act on local challenges, quality of care issues and health system obstacles. The project will be based on problem-solving cycles and study its effect on 388,000 births, to our knowledge the largest implementation study in this field. The PeriScope will make health systems more responsive and sustainable, reduce barriers currently preventing the uptake of safe, efficient and equitable perinatal health services. This project will allow for researcher with complimentary skills to embark on previous achievements and develop a strong collaboration. Lessons from this scale-up can be transferred to other settings, enabled by the active participation of the WHO, the UNICEF, and the World Bank.
2019	Sweden	Ethiopia	Reproductive health care	ODA Grants	0.0169984	Scaling Up Survive and Thrive Action Interventions (SUSTAIN).	The first 1,000 days of life, before and after birth, is the period with the most profound consequences for short- and long-term health. This period carries the burden of an annual 300,000 maternal deaths, 2,6 million stillbirths, and 4 million infant deaths. Furthermore, long-term consequences for the surviving but affected newborns include impaired cognitive development and increased risk of future chronic diseases. The period from onset of pregnancy to two years of age thus provides a unique window of opportunity to establish a solid foundation for survival, health, growth, and cognitive development.Low-income countries that were successful in the past few decades to expand primary health services and reduce child mortality now face new challenges. To further increase the chances for children to survive and thrive there is a need for communities to engage in the health and welfare of the mother-and-child dyad and primary health services to respond to these needs with quality services. There is well-researched evidence that the use of facilitated participatory groups is an effective social innovation but no large scale-up of this promising innovation has so far taken place.The current application aims to support the establishment of the multi-disciplinary SUSTAIN network that aim to develop and test social innovations

Year	Donor	Recipient	Purpose Code	Flow Type	Disbursement (million USD)	Project Title	Long Description
							across diverse health system settings to improve the health and survival during the first 1,000 days of life.
2019	Sweden	Rwanda	Reproductive health care	ODA Grants	0.0169984	Scaling Up Survive and Thrive Action Interventions (SUSTAIN).	The first 1,000 days of life, before and after birth, is the period with the most profound consequences for short- and long-term health. This period carries the burden of an annual 300,000 maternal deaths, 2,6 million stillbirths, and 4 million infant deaths. Furthermore, long-term consequences for the surviving but affected newborns include impaired cognitive development and increased risk of future chronic diseases. The period from onset of pregnancy to two years of age thus provides a unique window of opportunity to establish a solid foundation for survival, health, growth, and cognitive development.Low-income countries that were successful in the past few decades to expand primary health services and reduce child mortality now face new challenges. To further increase the chances for children to survive and thrive there is a need for communities to engage in the health and welfare of the mother-and-child dyad and primary health services to respond to these needs with quality services. There is well-researched evidence that the use of facilitated participatory groups is an effective social innovation but no large scale-up of this promising innovation has so far taken place.The current application aims to support the establishment of the multi-disciplinary SUSTAIN network that aim to develop and test social innovations across diverse health system settings to improve the health and survival during the first 1,000 days of life.

Year	Donor	Recipient	Purpose Code	Flow Type	Disbursement (million USD)	Project Title	Long Description
2018	Sweden	Viet Nam	Medical research	ODA Grants	0.0166851	Scaling up prevention of perinatal deaths. Implementation research on evidence-based participatory interventions across diverse health systems	The time before and after birth, the perinatal period, is the time of an annual 300,000 maternal deaths, 3 million stillbirths and 3 million newborn deaths. There is robust evidence that use of facilitated participatory groups is an effective social innovation to prevent a large proportion of these deaths to happen. No large scale-up of this promising innovation has so far taken place. The aim of this project is to adapt, implement, evaluate and communicate a contextually appropriate social innovation, the PeriScope model, for perinatal health in Viet Nam and Nepal representing low - high maternal and neonatal mortality. The project targets primary and secondary health system levels and engage stakeholder groups with more than 5,400 key members, health care staff and managers to identify and act on local challenges, quality of care issues and health system obstacles. The project will be based on problem-solving cycles and study its effect on 388,000 births, to our knowledge the largest implementation study in this field. The PeriScope will make health systems more responsive and sustainable, reduce barriers currently preventing the uptake of safe, efficient and equitable perinatal health services. This project will allow for researcher with complimentary skills to embark on previous achievements and develop a strong collaboration. Lessons from this scale-up can be transferred to other settings, enabled by the active participation of the WHO, the UNICEF, and the World Bank.
2018	Sweden	Nepal	Medical research	ODA Grants	0.0166851	Scaling up prevention of perinatal deaths. Implementation research on evidence-based participatory interventions across diverse health systems	The time before and after birth, the perinatal period, is the time of an annual 300,000 maternal deaths, 3 million stillbirths and 3 million newborn deaths. There is robust evidence that use of facilitated participatory groups is an effective social innovation to prevent a large proportion of these deaths to happen. No large scale-up of this promising innovation has so far taken place. The aim of this project is to adapt, implement, evaluate and communicate a contextually appropriate social innovation, the PeriScope model, for perinatal health in Vietnam and Nepal representing low - high maternal and neonatal mortality. The project targets primary and secondary health system levels and engage stakeholder groups with more than 5,400 key members, health care staff and managers to identify and act on local challenges, quality of care issues and health system obstacles. The project will be based on problem-solving cycles and study its effect on 388,000 births, to our knowledge the largest implementation study in this field. The PeriScope will make health systems more responsive and sustainable, reduce barriers currently preventing the uptake of safe, efficient and equitable perinatal health services. This project will allow for researcher with complimentary skills to embark on previous achievements and develop a

Year	Donor	Recipient	Purpose Code	Flow Type	Disbursement (million USD)	Project Title	Long Description
							strong collaboration. Lessons from this scale-up can be transferred to other settings, enabled by the active participation of the WHO, the UNICEF, and the World Bank.
2019	Sweden	Viet Nam	Medical research	ODA Grants	0.0162289	Scaling up prevention of perinatal deaths. Implementation research on evidence-based participatory interventions across diverse health systems	The time before and after birth, the perinatal period, is the time of an annual 300,000 maternal deaths, 3 million stillbirths and 3 million newborn deaths. There is robust evidence that use of facilitated participatory groups is an effective social innovation to prevent a large proportion of these deaths to happen. No large scale-up of this promising innovation has so far taken place. The aim of this project is to adapt, implement, evaluate and communicate a contextually appropriate social innovation, the PeriScope model, for perinatal health in Viet Nam and Nepal representing low - high maternal and neonatal mortality. The project targets primary and secondary health system levels and engage stakeholder groups with more than 5,400 key members, health care staff and managers to identify and act on local challenges, quality of care issues and health system obstacles. The project will be based on problem-solving cycles and study its effect on 388,000 births, to our knowledge the largest implementation study in this field. The PeriScope will make health systems more responsive and sustainable, reduce barriers currently preventing the uptake of safe, efficient and equitable perinatal health services. This project will allow for researcher with complimentary skills to embark on previous achievements and develop a strong collaboration. Lessons from this scale-up can be transferred to other settings, enabled by the active participation of the WHO, the UNICEF, and the World Bank.

Year	Donor	Recipient	Purpose Code	Flow Type	Disbursement (million USD)	Project Title	Long Description
2019	Sweden	Nepal	Medical research	ODA Grants	0.0162289	Scaling up prevention of perinatal deaths. Implementation research on evidence-based participatory interventions across diverse health systems	The time before and after birth, the perinatal period, is the time of an annual 300,000 maternal deaths, 3 million stillbirths and 3 million newborn deaths. There is robust evidence that use of facilitated participatory groups is an effective social innovation to prevent a large proportion of these deaths to happen. No large scale-up of this promising innovation has so far taken place. The aim of this project is to adapt, implement, evaluate and communicate a contextually appropriate social innovation, the PeriScope model, for perinatal health in Vietnam and Nepal representing low - high maternal and neonatal mortality. The project targets primary and secondary health system levels and engage stakeholder groups with more than 5,400 key members, health care staff and managers to identify and act on local challenges, quality of care issues and health system obstacles. The project will be based on problem-solving cycles and study its effect on 388,000 births, to our knowledge the largest implementation study in this field. The PeriScope will make health systems more responsive and sustainable, reduce barriers currently preventing the uptake of safe, efficient and equitable perinatal health services. This project will allow for researcher with complimentary skills to embark on previous achievements and develop a strong collaboration. Lessons from this scale-up can be transferred to other settings, enabled by the active participation of the WHO, the UNICEF, and the World Bank.
2019	Sweden	Malawi	Reproductive health care	ODA Grants	0.0149231	Safe and respectful maternity care: Setting up a network for research in tertiary hospitals	At least two million lives are lost every year globally due to complications in the intrapartum period. We aim to initiate research to prevent this high number of stillbirths and neonatal deaths by forming a consortium of researchers working in large referral hospitals in East Africa. We aim to define and innovate interventions and perform implementation research focused on the recently published new intrapartum care guidelines of the World Health Organisations. Respectful intrapartum care shall be in the centre, and we will assess the potential of lactate point-of-care testing to diagnose hypoxia and fetal monitoring using the new Moyo Fetal Heart Rate Monitor.Network partners are Makerere University and Iganga hospital, Uganda, Muhimbili University Hospital and Aga Khan University, Tanzania, the College of Medicine in Blantyre, Malawi and Hospital Central Maputo, Mozambique. During the two-year period for which we apply for funding we aim to 1) innovate implementation packages to improve the provision of safe and respectful intrapartum care; 2) review present systems of perinatal data collection or e-registries in identified hospitals 3; investigate into the feasibility of improved diagnosis of peripartum hypoxia using umbilical cord lactate measurement; and 4) prepare jointly research questions and

Year	Donor	Recipient	Purpose Code	Flow Type	Disbursement (million USD)	Project Title	Long Description
							applications. This network is primarily targeting the funding opportunity of the upcoming implementation science call for maternal and child health of Horizon 2020.
2019	Sweden	Tanzania	Reproductive health care	ODA Grants	0.0149231	Safe and respectful maternity care: Setting up a network for research in tertiary hospitals	At least two million lives are lost every year globally due to complications in the intrapartum period. We aim to initiate research to prevent this high number of stillbirths and neonatal deaths by forming a consortium of researchers working in large referral hospitals in East Africa. We aim to define and innovate interventions and perform implementation research focused on the recently published new intrapartum care guidelines of the World Health Organisations. Respectful intrapartum care shall be in the centre, and we will assess the potential of lactate point-of-care testing to diagnose hypoxia and fetal monitoring using the new Moyo Fetal Heart Rate Monitor.Network partners are Makerere University and Iganga hospital, Uganda, Muhimbili University Hospital and Aga Khan University, Tanzania, the College of Medicine in Blantyre, Malawi and Hospital Central Maputo, Mozambique. During the two-year period for which we apply for funding we aim to 1) innovate implementation packages to improve the provision of safe and respectful intrapartum care; 2) review present systems of perinatal data collection or e-registries in identified hospitals 3; investigate into the feasibility of improved diagnosis of peripartum hypoxia using umbilical cord lactate measurement; and 4) prepare jointly research questions and applications. This network is primarily targeting the funding opportunity of the upcoming implementation science call for maternal and child health of Horizon 2020.

Year	Donor	Recipient	Purpose Code	Flow Type	Disbursement (million USD)	Project Title	Long Description
2019	Sweden	Uganda	Reproductive health care	ODA Grants	0.0149231	Safe and respectful maternity care: Setting up a network for research in tertiary hospitals	At least two million lives are lost every year globally due to complications in the intrapartum period. We aim to initiate research to prevent this high number of stillbirths and neonatal deaths by forming a consortium of researchers working in large referral hospitals in East Africa. We aim to define and innovate interventions and perform implementation research focused on the recently published new intrapartum care guidelines of the World Health Organisations. Respectful intrapartum care shall be in the centre, and we will assess the potential of lactate point-of-care testing to diagnose hypoxia and fetal monitoring using the new Moyo Fetal Heart Rate Monitor.Network partners are Makerere University and Iganga hospital, Uganda, Muhimbili University Hospital and Aga Khan University, Tanzania, the College of Medicine in Blantyre, Malawi and Hospital Central Maputo, Mozambique. During the two-year period for which we apply for funding we aim to 1) innovate implementation packages to improve the provision of safe and respectful intrapartum care; 2) review present systems of perinatal data collection or e-registries in identified hospitals 3; investigate into the feasibility of improved diagnosis of peripartum hypoxia using umbilical cord lactate measurement; and 4) prepare jointly research questions and applications. This network is primarily targeting the funding opportunity of the upcoming implementation science call for maternal and child health of Horizon 2020.
2017	Germany	Benin	Basic health infrastructure	ODA Grants	0.00263155	Support of the medical stations around Bassila and the hospital in Bassila	Acquisition of medical equipment to examine women during pregnancy and birth, local staff training. These arragements can minimalize the rate of stillbirthes, miscarriages and complication during birthes. Also it enables a sustainable chance to improve the situation of local socially disadvantaged people and to support their selfhelp.

S6: Absolute value of global aid mentioning newborns and stillbirths, by CRS purpose code, 2002-19

Purpose Code	Purpose	Exclusively NBSB	Includes NBSB and others	Total	% Total
11110	Education policy and administrative management	0.0	4.5	4.5	0.0%
11120	Education facilities and training	0.1	0.0	0.1	0.0%
11130	Teacher training	0.0	0.2	0.2	0.0%
11220	Primary education	0.0	1.6	1.6	0.0%
11230	Basic life skills for youth and adults	0.0	10.1	10.1	0.1%
11240	Early childhood education	0.0	1.2	1.2	0.0%
11420	Higher education	1.2	2.0	3.3	0.0%
11430	Advanced technical and managerial training	0.0	0.1	0.1	0.0%
12110	Health policy and administrative management	3.2	398.7	401.9	3.3%
12181	Medical education/training	2.2	35.6	37.8	0.3%
12182	Medical research	61.5	172.3	233.7	1.9%
12191	Medical services	0.9	221.7	222.6	1.8%
12220	Basic health care	37.6	1158.9	1196.5	9.7%
12230	Basic health infrastructure	3.7	200.7	204.4	1.7%
12240	Basic nutrition	4.5	622.8	627.3	5.1%
12250	Infectious disease control	42.8	438.6	481.4	3.9%
12261	Health education	0.0	91.1	91.1	0.7%
12262	Malaria control	0.0	248.6	248.6	2.0%
12263	Tuberculosis control	0.3	186.1	186.3	1.5%
12281	Health personnel development	0.0	186.7	186.7	1.5%
12340	Promotion of mental health and well-being	0.0	0.1	0.1	0.0%
13010	Population policy and administrative management	1.2	82.7	84.0	0.7%
13020	Reproductive health care	315.3	5359.8	5675.1	45.9%
13030	Family planning	9.6	735.1	744.7	6.0%
13040	STD control including HIV/AIDS	25.6	1021.7	1047.3	8.5%
13081	Personnel development for population and reproductive health	6.4	268.9	275.3	2.2%
14010	Water sector policy and administrative management	0.0	9.4	9.4	0.1%
14020	Water supply and sanitation - large systems	0.0	11.8	11.8	0.1%
14021	Water supply - large systems	0.0	0.3	0.3	0.0%
14030	Basic drinking water supply and basic sanitation	0.0	36.7	36.7	0.3%
14031	Basic drinking water supply	0.0	24.8	24.8	0.2%
14032	Basic sanitation	0.0	35.4	35.4	0.3%
14050	Waste management/disposal	0.0	0.3	0.3	0.0%
14081	Education and training in water supply and sanitation	0.0	12.9	12.9	0.1%
15110	Public sector policy and administrative management	0.0	4.3	4.3	0.0%
15111	Public finance management (PFM)	0.0	0.1	0.1	0.0%
15112	Decentralisation and support to subnational government	0.0	3.7	3.7	0.0%
15114	Domestic revenue mobilisation	0.0	0.7	0.7	0.0%
15150	Democratic participation and civil society	0.0	1.2	1.2	0.0%
15153	Media and free flow of information	0.0	0.1	0.1	0.0%
15160	Human rights	0.0	3.8	3.8	0.0%
15170	Women's rights organisations and movements, and government institutions	0.0	0.1	0.1	0.0%
15180	Ending violence against women and girls	0.0	4.8	4.8	0.0%
15210	Security system management and reform	0.0	0.1	0.1	0.0%
16010	Social Protection	0.1	25.2	25.2	0.2%
16050	Multisector aid for basic social services	0.4	15.5	15.9	0.1%
16064	Social mitigation of HIV/AIDS	0.0	4.0	4.0	0.0%
22010	Communications policy and administrative management	0.0	0.5	0.5	0.0%
23310	Energy generation, non-renewable sources, unspecified	0.0	0.4	0.4	0.0%
24081	Education/training in banking and financial services	0.0	1.2	1.2	0.0%
25010	Business policy and administration	0.0	0.7	0.7	0.0%
25020	Privatisation	0.0	1.5	1.5	0.0%
31110	Agricultural policy and administrative management	0.0	2.0	2.0	0.0%
31120	Agricultural development	0.0	18.9	18.9	0.2%
31150	Agricultural inputs	0.0	0.0	0.0	0.0%
31182	Agricultural research	0.0	0.0	0.0	0.0%
31382	Fishery research	0.1	0.0	0.1	0.0%
32130	Small and medium-sized enterprises (SME) development	0.0	0.0	0.0	0.0%
32161	Agro-industries	0.0	0.1	0.1	0.0%
33110	Trade policy and administrative management	0.0	0.0	0.0	0.0%
41010	Environmental policy and administrative management	0.0	4.2	4.2	0.0%
43010	Multisector aid	0.1	8.9	9.1	0.1%
43030	Urban development and management	0.0	2.7	2.7	0.0%
43040	Rural development	0.0	4.0	4.0	0.0%
43081	Multisector education/training	0.0	1.3	1.3	0.0%
43082	Research/scientific institutions	0.0	1.3	1.3	0.0%
72010	Material relief assistance and services	5.4	76.1	81.5	0.7%
72040	Emergency food assistance	0.0	0.3	0.3	0.0%
72050	Relief co-ordination and support services	0.0	18.8	18.8	0.2%
73010	Immediate post-emergency reconstruction and rehabilitation	1.5	3.9	5.4	0.0%
91010	Administrative costs (non-sector allocable)	0.0	1.3	1.3	0.0%
93010	Refugees/asylum seekers in donor countries (non-sector allocable)	0.0	0.1	0.1	0.0%
99810	Sectors not specified	5.6	4.8	10.4	0.1%
99820	Promotion of development awareness (non-sector allocable)	0.0	27.6	27.6	0.2%
**Total**		**529.4**	**11826.0**	**12355.4**	**100.0%**

S7: Global aid mentioning newborns and stillbirths by donor, 2002-19

Legend: In Figure 3, donors are ranked in descending order by aid including NB/SB provided during the period 2002-2019 (including nb/sb shown in yellow; nb/sb focused shown in orange). This is contextualized against the Muskoka2 estimates for maternal and newborn health (one category) as shown in grey. NB in the case of Canada, the estimates from Muskoka2 for MNH were less than the estimated value of aid benefitting NB/SB, hence the negative values shown in the figure.

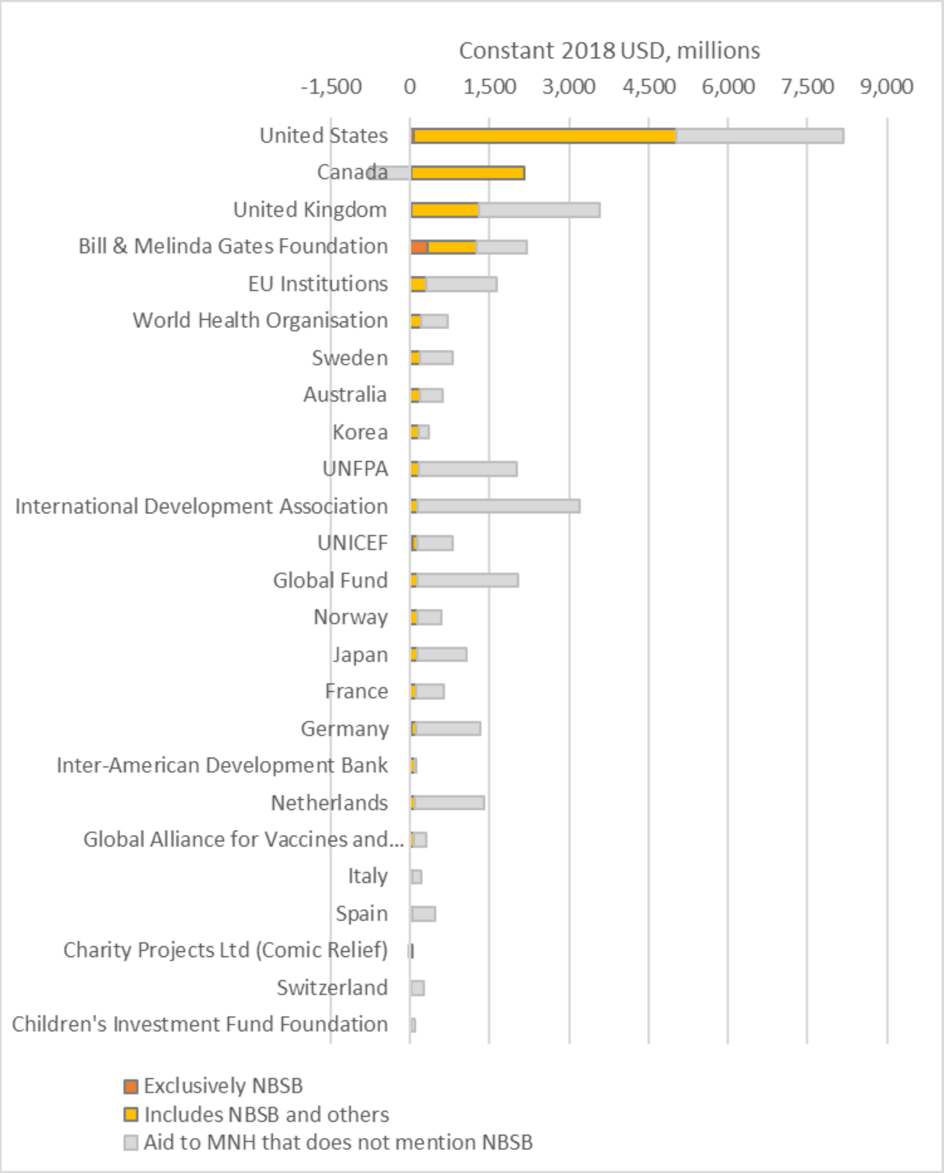

S8: Value of aid by donor / by year for top donors (million 2019USD)

Year	United States	Canada	Bill & Melinda Gates Foundation	United Kingdom	EU Institutions	Sweden	UNICEF	Australia	Other donors
2002	0.0	2.0	0.0	2.0	0.0	0.4	3.7	0.2	11.3
2003	0.0	24.3	0.0	2.8	0.0	0.1	2.2	0.7	10.6
2004	0.0	21.7	0.0	2.0	0.0	0.0	5.5	0.4	3.0
2005	12.2	19.4	0.0	4.7	0.0	6.6	6.8	0.5	2.2
2006	8.1	18.2	0.0	2.0	0.0	0.3	4.6	0.2	11.3
2007	36.8	31.5	0.0	8.3	0.6	0.1	6.2	0.2	14.5
2008	224.5	27.5	0.0	41.2	4.6	0.7	9.8	7.9	28.6
2009	451.8	51.6	121.7	9.2	5.5	1.7	7.1	9.5	31.1
2010	331.8	76.9	105.3	13.2	5.7	2.5	6.7	13.8	201.9
2011	373.0	248.8	136.3	37.2	7.7	8.8	9.5	19.7	75.2
2012	404.3	236.3	132.0	167.7	28.3	52.2	9.4	61.7	77.4
2013	462.0	236.6	139.5	131.2	32.6	15.1	8.0	49.2	104.2
2014	455.8	367.3	143.8	172.5	51.3	8.6	15.3	48.0	107.6
2015	545.1	358.9	175.1	169.2	80.0	9.0	22.9	23.7	145.2
2016	523.0	390.6	140.8	191.8	77.1	30.1	23.5	5.8	133.9
2017	550.0	508.1	190.4	220.9	37.0	57.7	22.5	8.8	135.1
2018	505.5	108.1	181.2	232.0	6.7	69.2	20.9	2.0	156.8
2019	410.0	366.6	151.3	180.7	21.8	48.3	120.5	0.7	188.1
	**5,293.9**	**3,094.4**	**1,617.5**	**1,588.7**	**359.1**	**311.2**	**305.1**	**253.0**	**1,438.0**

S9. Absolute value of global aid mentioning newborns and stillbirths by recipient in the context of Muskoka2 MNH estimates, 2002-19

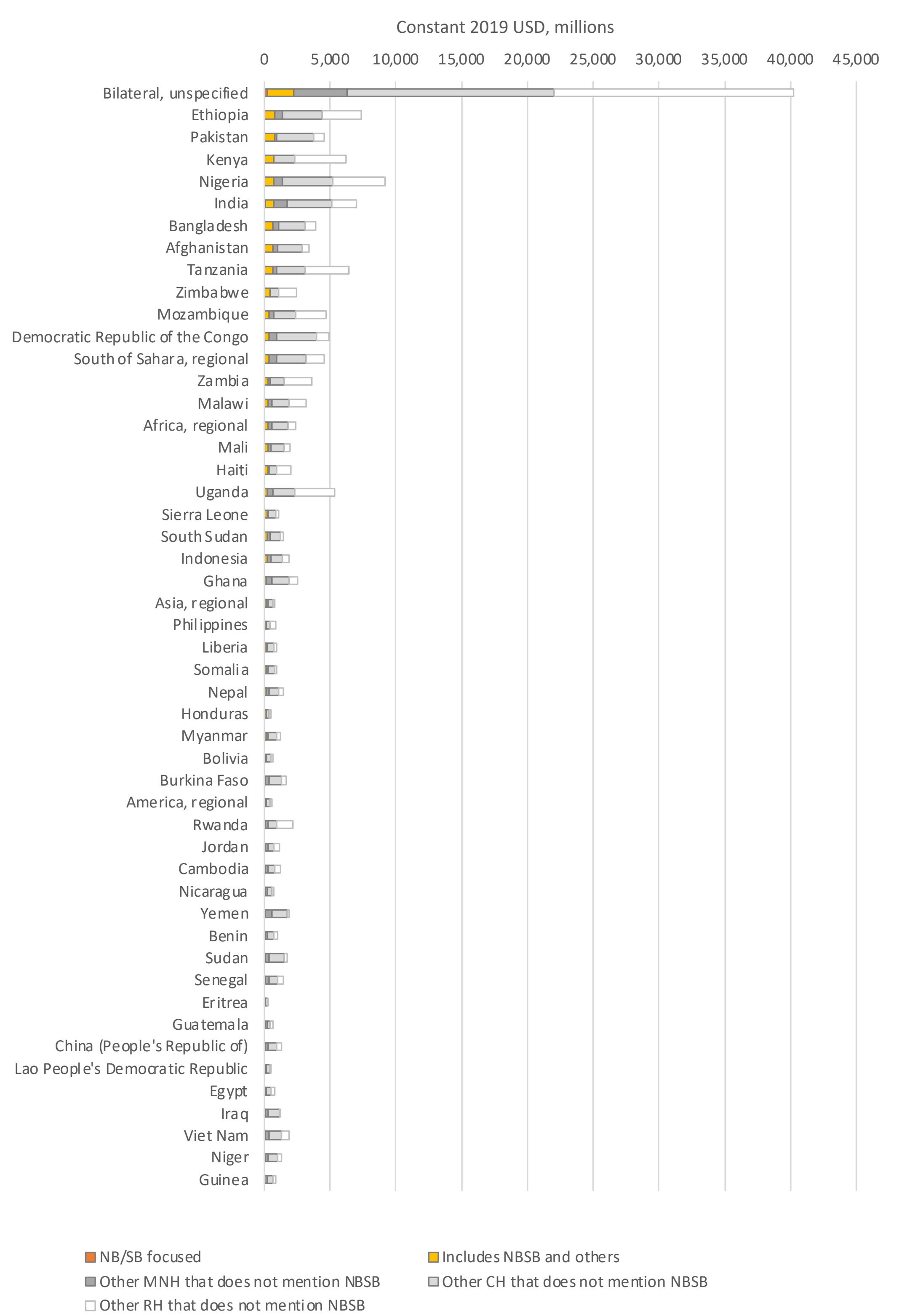

S10. Number of neonatal deaths and stillbirths and amount of aid received in Least Developed Countries, 2002-2019

Country	stillbirths 2002 -2019	newborn deaths 2002 - 2019	total stillbirths and newborn deaths 2002 - 2019	NB/SB-focused ODA 2002 -19	ODA including NB/SB 2002-19	ODA per death
Bhutan	3014	5442	8456	0.23452003	2	$ 251.47
Timor-Leste	10393	17333	27726	0.05866087	38	$ 1,389.74
Lesotho	33602	39598	73200	0.00405242	18	$ 245.76
Eritrea	35261	40684	75945	0.67686089	60	$ 801.82
Gambia	34764	45768	80532	1.20114892	21	$ 277.13
Guinea-Bissa	43344	48148	91492	0.0618988	21	$ 231.03
Congo	51273	66072	117345	0.00588315	2	$ 20.78
Lao PDR	61158	88089	149247	0.58453804	58	$ 395.10
Liberia	69652	93104	162756	0.0118718	169	$ 1,037.05
CAR	101612	128388	230000	0.18596227	37	$ 159.98
Haiti	96398	135440	231838	0.18679117	274	$ 1,183.50
Cambodia	103099	129909	233008	0.55405207	93	$ 403.44
Rwanda	133103	160003	293106	1.0421011	103	$ 354.98
Sierra Leone	125553	175121	300674	0.0018179	238	$ 791.03
Burundi	188196	194800	382996	0.0515968	31	$ 80.72
Benin	166262	230578	396840	0.39730304	68	$ 171.20
Mauritania	358754	64650	423404	0.15540586	10	$ 23.43
Guinea	190295	261173	451468	0.01337291	46	$ 101.67
Zambia	181515	280822	462337	12.3875408	310	$ 696.87
South Sudan	186850	290453	477303	0	234	$ 490.33
Malawi	203892	276149	480041	14.3184252	285	$ 623.60
Nepal	271316	304943	576259	10.1970119	143	$ 265.97
Madagascar	267212	357223	624435	0.12288776	43	$ 69.06
Burkina Faso	292985	386472	679457	0.14510886	116	$ 171.26
Chad	301662	391548	693210	0.00951383	36	$ 52.03
Somalia	338100	443331	781431	0.51676948	155	$ 199.26
Myanmar	309660	502812	812472	0.6473659	146	$ 180.28
Yemen	366959	475745	842704	0.06448662	74	$ 87.86
Niger	339679	527967	867646	0.89099541	49	$ 57.22
Mali	358754	517763	876517	6.02541196	282	$ 328.90
Mozambique	356233	593062	949295	3.68046356	393	$ 417.82
Angola	437077	682371	1119448	0.33480591	21	$ 18.84
Uganda	514859	627664	1142523	3.5908407	237	$ 210.39
Sudan	570766	714649	1285415	0.00769013	66	$ 51.58
Afghanistan	675154	992771	1667925	0.40031055	662	$ 397.32
DRC	1661503	1741560	3403063	0.33071614	372	$ 109.45
Bangladesh	1864892	1740967	3605859	19.8479826	669	$ 190.93
Ethiopia	1667349	2210676	3878025	17.6260093	804	$ 211.74
